# Systemic IFN-I combined with topical TLR7/8 agonists promotes distant tumor suppression by c-Jun-dependent IL-12 expression in dendritic cells

**DOI:** 10.1038/s43018-024-00889-9

**Published:** 2025-01-23

**Authors:** Martina Sanlorenzo, Philipp Novoszel, Igor Vujic, Tommaso Gastaldi, Martina Hammer, Ourania Fari, Cristiano De Sa Fernandes, Alina D. Landau, Bilge V. Göcen-Oguz, Martin Holcmann, Babak Monshi, Klemens Rappersberger, Agnes Csiszar, Maria Sibilia

**Affiliations:** 1 Center for Cancer Research, Medical University of Vienna, Comprehensive Cancer Center, Vienna, Austria; 2 Department of Dermatology, Klinik Landstrasse, Vienna, Austria

**Keywords:** Melanoma, Toll-like receptors, Cancer immunotherapy, Cancer

## Abstract

Dendritic cell (DC) activation by pattern recognition receptors like Toll-like-receptors (TLRs) is crucial for cancer immunotherapies. Here, we demonstrate the effectiveness of the TLR7/8 agonist imiquimod (IMQ) in treating both local tumors and distant metastases. Administered orally, IMQ activates plasmacytoid DCs (pDCs) to produce systemic type I interferons (IFN-I) required for TLR7/8 upregulation in DCs and macrophages, sensitizing them to topical IMQ treatment, which is essential for therapeutic efficacy. The mechanism involves c-Jun/AP-1 mediating TLR7/8 signaling in IFN-I-primed DCs, upregulating the pDC-recruiting chemokine CCL2 and the anti-angiogenic cytokine interleukin-12, which suppresses VEGF-A production leading to tumor necrosis and regression. Combining topical and systemic IMQ or IFN-I generates a CD8^+^ T cell-dependent response at metastatic sites, reinforced by PD-1 blockade, leading to long-lasting memory. Analysis of cohorts of patients with melanoma demonstrates DC-specific TLR7/8 upregulation by IFN-I, supporting the translational potential of combining systemic IFN-I and topical IMQ to improve immunotherapy of topically accessible tumors.

## Abstract (web summary)

Sanlorenzo et al. propose a strategy to boost immunotherapy by combining topically applied TLR7/8 agonists with systemic interferon-I, sensitizing dendritic cells to produce IL-12 for tumor clearance and an adaptive immune response at metastatic sites.

## Main

Cancer immunotherapy induces different immune responses in patients and has attracted substantial interest due to its potential for treating a wide range of human cancers^1^. Unfortunately, immunotherapies are still not effective in many patients and other strategies are being investigated.

TLRs and their agonists represent a powerful way to induce antitumor immune responses. They are potent immune response modifiers that recognize diverse molecular patterns found on pathogens. Through their activation, TLRs play a pivotal role in stimulating both the innate and adaptive arms of the immune system^2^. TLR7/8 detects viral or self-RNA, and its activation by a TLR7 agonist, such as IMQ^3^ can elicit robust antitumor immune responses. TLR7/8 agonists are already utilized in clinical settings for the treatment of actinic keratosis^4^ and basal cell carcinoma^5^.

pDCs are a subset of TLR7/8-expressing DCs that rapidly produce IFN-I upon stimulation^6^. We previously showed that pDCs are crucial for the antitumor effect of IMQ in preclinical models of melanoma. Topical treatment with IMQ recruits pDCs to the tumor site via the chemokine CCL2 and converts them into tumor-killing effector cells, leading to tumor shrinkage at the treatment site^7^. Of note, adaptive immune cells (T and B cells) and natural killer (NK) cells were not required for the antitumor effect of IMQ at the primary site^7^.

Although pDCs reside in a variety of healthy tissues^8^, they are rarely found in the skin and within the tumor microenvironment (TME) of skin cancers, like melanoma^9^. After topical IMQ administration, pDCs are recruited to the skin 1–2 days after treatment^10^, whereas IFN-I is induced already 1–3 h after treatment^3,11^. Therefore, one unresolved question is how and by which cells IFN-I is produced at the beginning of topical IMQ treatment. Given the low abundance of pDCs in the TME, it seems unlikely that they are the primary source of IFN-I.

It is possible that in mice, topical IMQ application could lead to unintended systemic activation of pDCs possibly through oral uptake due to grooming behavior. Indeed, in a mouse psoriasis-like skin model, it has been shown that the use of Elizabethan collars, which prevent ingestion, attenuates the skin inflammation promoted by topical IMQ^12^. Furthermore, although administered topically, some preclinical studies suggest that TLR7/8 agonists lead to a systemic immune response that is effective also on distant, nontreated metastases^13^ and intravenous delivery of TLR7/8 agonists has been shown to elicit an antitumor CD8^+^ T cell immune response^14^.

Despite the wealth of data accumulated over the past decades, still little is known about the spatial and temporal activity of IMQ and the underlying molecular antitumor mechanism. In particular, the cellular networks driving antitumor immunity of IMQ in pDCs and other DC subsets remain elusive. c-Jun is a member of the AP-1 family of transcription factors and has previously been reported to act downstream of TLR7 signaling in DCs to promote IMQ-induced psoriasis-like skin inflammation^10^; however, the role of c-Jun/AP-1 in DC-mediated antitumor immunity has not been addressed.

Here, we used genetically engineered mouse models, preclinical tumor models and human melanoma samples to uncover a previously unexplored role of pDCs and DCs in the antitumor immune response induced by IMQ. Specifically, we demonstrate that pDCs play a crucial role in the systemic production of IFN-I in response to oral IMQ treatment. This induction of IFN-I leads to the upregulation of TLR7/8 expression on DCs and macrophages within the TME. At the topical tumor site, local immune activation by IMQ stimulates c-Jun/AP-1 signaling in IFN-I primed and TLR7-expressing DCs to produce interleukin (IL)-12, which impairs tumor-associated angiogenesis and induces necrosis at the treated site. This therapeutic strategy also demonstrates remarkable efficacy in preventing tumor metastasis and relapse by promoting the formation of CD8^+^ T effector/memory cells. Moreover, we show that this combination therapy sensitizes melanoma tumors to checkpoint inhibitor treatment with enhanced memory formation. Thus, our results could have significant implications for the treatment of patients with topically accessible tumors like melanoma or breast tumors and could help in improving patient outcomes.

## Results

### Oral and topical IMQ promotes local and distant effects

**Extended Data Fig. 1:**
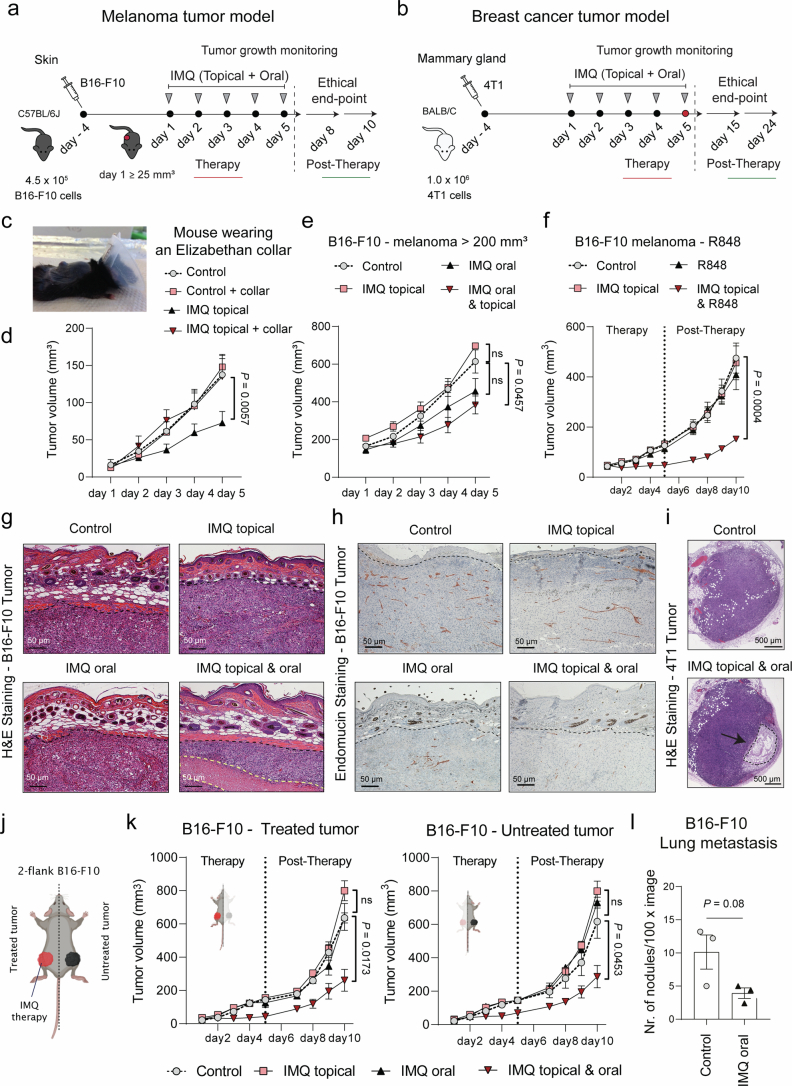
Oral and topical IMQ therapy induces tumor regression with necrosis. **a, b**, Scheme of the murine orthotopic tumor models employed. B16-F10 (a) or 4T1 (b) tumor cells were intradermally injected. IMQ was administered for five consecutive days (Therapy). Tumors were monitored after treatment (Post-Therapy). **c**, Representative picture of a tumor-bearing mouse wearing a collar. **d, e**, B16-F10 tumor growth curves in mice treated with IMQ, comparing mice with or without an Elizabethan collar (d) (*n* = 7 mice per group and in the Control group *n* = 6) or in mice where IMQ treatment was started when tumors had a size of 200 mm³ (e). (Control *n* = 5, IMQ: oral *n* = 3, topical *n* = 3, topical &oral *n* = 5). *n* is the number of mice pooled from (d) 2 independent experiments or from 1 experiment (e). **f**, Tumor growth (B16-F10) was monitored in mice treated topically with IMQ, orally with R848, or with a combination of both. (Control *n* = 4, R848 *n* = 3, IMQ: topical *n* = 3, topical &R848 *n* = 4; *n* is the number of mice from 1 experiment). **g**, Representative pictures of B16-F10 tumors (Fig. 1e) stained with hematoxylin and eosin. The black-dotted line demarcates the tumor boarder and the yellow line the necrotic area. Magnification: 10x. Scale bar: 50 µm. **h**, Immunohistochemistry staining of Endomucin in B16-F10 tumors treated as indicated, and described in (Fig. 1f). Magnification: 10x. Scale bar: 50 µm. **i**, Representative pictures of 4T1 breast cancer tumors (Fig. 1g) stained with hematoxylin and eosin. The arrow indicates the necrotic area. Magnification: 4x. Scale bar: 500 µm. **j**, 2-flank tumor model. B16-F10 cells were injected in both flanks. (left: Treated and right: Untreated). **k**, Tumor volume for treated (left graph) and untreated (right graph) B16-F10 tumors in a 2-flank B16-F10 tumor model described in (j). (Control *n* = 3, IMQ: oral *n* = 3, topical *n* = 3, topical & oral *n* = 5; *n* is the number of mice from 1 experiment). **l**, Quantification of lung metastasis in B16-F10 melanoma-bearing mice as described in (Fig. 1i). (*n* = 3 mice per group from 1 experiment). Data are shown as mean ± SEM. Dots in l represent biological replicates. *P*‐values were calculated using unpaired, two-tailed *t*-test (l) and two-way ANOVA with Tukey´s (d–f) or Dunnett´s post-test (k). Source data

To explore the local and distant antitumor potential of the TLR7/8 agonist IMQ, we employed orthotopic and syngeneic melanoma and breast cancer models (Extended Data Fig. 1a,b), as such tumors are easily accessible in patients for topical treatment and often form distant metastases^15^.

**Fig. 1:**
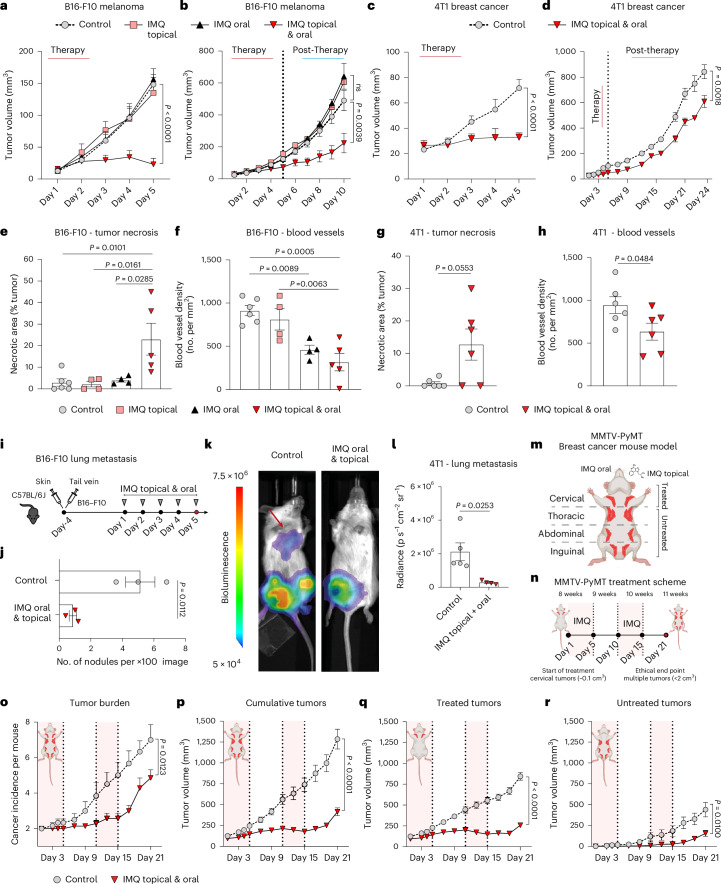
Oral and topical IMQ promotes local and distant antitumor effects. **a**–**d**, Tumor growth curves of mice treated with IMQ orally, topically or both for five consecutive days (therapy; see Extended Data Fig. 1a,b). All mice wore a collar. After treatment termination tumor growth was monitored to the ethical end point (post-therapy). B16-F10 melanoma growth under therapy (control *n* = 7, IMQ: topical *n* = 7, oral *n* = 8, topical and oral *n* = 8) (**a**) and post-therapy (control *n* = 5, IMQ: topical *n* = 4, oral *n* = 3, topical and oral *n* = 5) (**b**). 4T1 breast cancer under therapy (*n* = 6 mice per group) (**c**) and post-therapy (control *n* = 5, IMQ: topical and oral *n* = 4) (**d**). *n* in **a**–**d** is the number of mice pooled from two (**a**,**c**) independent experiments and from one experiment (**b**,**d**). **e**,**f**, Quantification of necrotic areas (**e**) in H&E-stained sections of B16-F10 tumors from **a** and of blood vessels (**f**) in endomucin-stained sections. Control *n* = 6, IMQ: topical *n* = 4, oral *n* = 4, topical and oral *n* = 5; *n* is the number of mice pooled from two independent experiments. **g**,**h**, Tumor necrosis (**g**) and blood vessels (**h**) were analyzed in 4T1 breast cancers (**c**). *n* = 6 mice per group pooled from two independent experiments. **i**, B16-F10 lung metastasis model. Intradermal and tail vein injections of B16-F10 tumor cells were performed. IMQ was administered topically, on the primary tumor and orally. **j**, Quantification of B16-F10-lung metastasis from **i**. *n* = 3 mice per group from one experiment. **k**, Lung metastasis was assessed by bioluminescence in 4T1 breast cancer-bearing mice from **d** 24 days after treatment start. **l**, Quantification of 4T1-lung metastasis from **k**. Control *n* = 5, IMQ topical and oral *n* = 4; *n* is the number of mice from one experiment. **m**,**n**, MMTV-PyMT breast cancer mouse model (**m**). Female mice received combination therapy at 8 weeks of age (first palpable tumors). IMQ was administered orally and topically on the breast (5 days), followed by a second round after a 5-day break (**n**). **o**–**r**, Tumor burden (**o**) and tumor growth curves showing cumulative (**p**), treated (**q**) and untreated (**r**) tumor sizes. In **o**–**r**, control *n* = 6, IMQ topical and oral *n* = 7; *n* is the number of mice from one experiment. Data are plotted as mean ± s.e.m. Dots represent biological replicates (**e**–**h**,**j**,**l**). *P* values were calculated by unpaired, two-tailed *t*-test (**j**) with Welch’s correction (**l**), one-way ANOVA (**e**–**h**) and two-way ANOVA with Tukey’s post-test (**a**–**d**,**o**–**r**). Source data

We previously reported that treatment of the primary tumor with IMQ blocks local B16-F10 melanoma growth in mice^7^. Here, we additionally used Elizabethan collars on mice to prevent unintended ingestion of the drug through grooming allowing us to distinguish between local (topical) and systemic effects (Extended Data Fig. 1c). Notably, the collars did not impact tumor growth in mice (Extended Data Fig. 1d). After topical IMQ treatment, only mice without collar showed reduced tumor growth, whereas the IMQ effect was lost in mice wearing the collar suggesting that the oral ingestion of IMQ was necessary for its tumoricidal activity (Extended Data Fig. 1d). But, also oral administration of IMQ alone did not have an antitumor effect and only concomitant oral and topical IMQ treatment was effective (Fig. 1a,b). Similarly, in the 4T1 breast cancer model combined oral and topical IMQ administration strongly inhibited tumor growth (Fig. 1c,d). A weaker but still significant antitumor effect of combined topical and oral IMQ treatment persisted following treatment discontinuation in both melanoma and breast cancer models (Fig. 1b–d). In large tumors (tumor size >200 mm³), the antitumor effect of the therapy was also weaker compared to controls, but still significant (Extended Data Fig. 1e). We also found that the therapeutic success was not limited to the TLR7 agonist IMQ, as a different TLR7/8 agonist (Resiquimod, R848) was also effective in melanoma (Extended Data Fig. 1f).

Histological examination of B16-F10 melanoma and 4T1 breast cancer tumors after topical and oral IMQ treatment revealed extensive necrotic areas and reduced numbers of blood vessels in the tumors, indicating that some of the antitumor activity of IMQ was due to its anti-angiogenic effects (Fig. 1e–h and Extended Data Fig. 1g–i). Notably, melanomas treated with topical IMQ alone did not show tumor necrosis (Fig. 1e), suggesting that the observed local, necrotic effect is not a result of drug absorption alone. These data show that combination therapy with IMQ topical and oral has potent antitumor effects in accessible tumors.

We next characterized the effects of our therapeutic approach on distant (non-accessible) tumors. In a two-flank tumor model, where B16-F10 melanoma were induced in both flanks (Extended Data Fig. 1j), we observed antitumor effects on both the treated tumor and untreated tumor (contralateral flank of treated), when we used the combined IMQ treatment (Extended Data Fig. 1k). Furthermore, concomitant oral and topical IMQ therapy also prevented lung metastases in a model that mimics lung melanoma metastases^16^ (Fig. 1i,j and Extended Data Fig. 1l) and in the 4T1 breast cancer model, which spontaneously gives lung metastases (Fig. 1k–l).

We also validated the efficacy of our therapeutic regimen in an autochthonous genetically engineered mouse model of breast cancer (MMTV-PyMT). At 8 weeks of age, when palpable tumors had developed in the mammary gland (Fig. 1m), tumors located in the cervical mammary fat pad were treated with combined topical and oral IMQ for 5 days, followed by an additional 5 days of IMQ treatment after a 5-day break (Fig. 1n). The total tumor burden and the cumulative tumor volume was significantly reduced in treated mice (Fig. 1o–p). Of note, the topically untreated tumors located in the thoracic, abdominal and inguinal mammary fat pad were also overall reduced in size, similar to the treated mammary tumors (Fig. 1q–r), suggesting that IMQ treatment is effective also at distant sites.

Together, these results indicate that the antitumor effect of IMQ is not restricted to topically accessible tumors but extends also to distant and metastatic sites.

### The antitumor effect of IMQ depends on pDCs and type I IFN

**Fig. 2:**
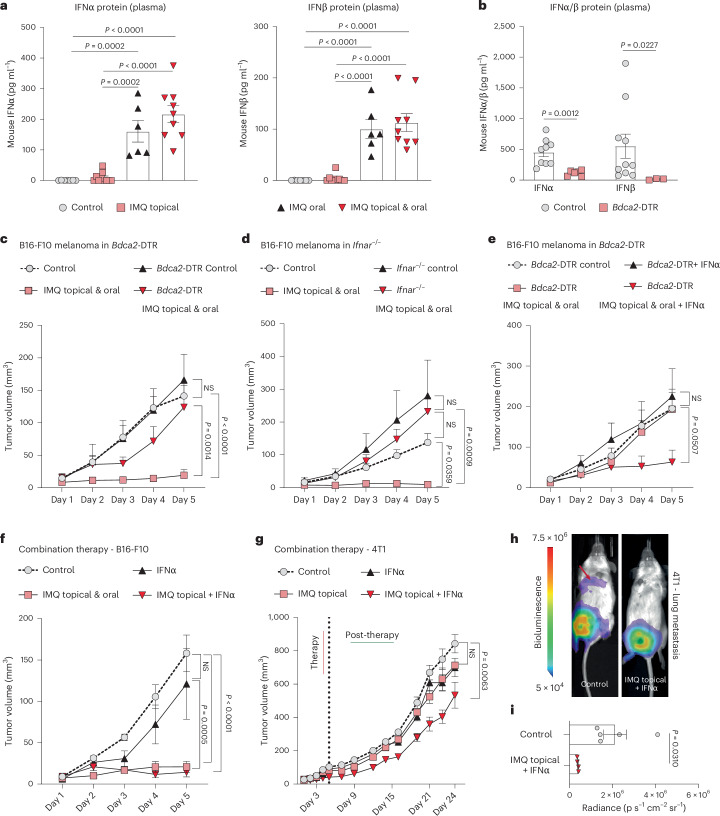
The antitumor effect of IMQ depends on pDCs and type I IFN. **a**,**b**, Protein levels of IFNα and IFNβ (**a**) in the plasma of mice 1 h after treatment with topical IMQ, oral IMQ, and topical plus oral IMQ in wild-type mice (control *n* = 7, IMQ: topical *n* = 10, oral *n* = 8, topical and oral *n* = 9) and in and mice depleted of pDCs (*Bdca2*-DTR) (**b**). IFNα: control *n* = 9, *Bdca2*-DTR *n* = 6, IFNβ: control *n* = 10, *Bdca2*-DTR *n* = 3; *n* corresponds to the number of individual mouse plasma pooled from two independent experiments. **c**, Tumor growth kinetics (B16-F10) were assessed in mice depleted of pDCs (*Bdca2*-DTR). The mice were treated with topical and oral IMQ. Control *n* = 4, IMQ topical and oral *n* = 7*, Bdca2*-DTR control *n* = *6*, IMQ topical and oral *n* = 8; *n* is the number of mice pooled from two independent experiments. **d**–**f** Tumor growth (B16-F10) was monitored in mice lacking the type I IFN receptor (*Ifnar*^−/^^−^) (*n* = 6 mice per group pooled from two independent experiments) (**d**), in mice depleted of pDCs (*Bdca2*-DTR) (*Bdca2*-DTR control *n* = 4, IFNα *n* = 6, IMQ topical and oral *n* = 6, IMQ topical and oral + IFNα *n* = 6; *n* is the number of mice pooled from two independent experiments) (**e**) and in wild-type mice (control *n* = 6, IFNα *n* = 6, IMQ topical and oral *n* = *5*, IMQ topical + IFNα *n* = *5*; *n* is the number of mice pooled from two independent experiments) (**f**). **g**, Tumor growth was monitored in 4T1 breast cancer-bearing mice during therapy (5 days) and after treatment termination (post-therapy). Therapy included treatment with IFNα and/or IMQ topically. Control and IMQ topical + IFNα *n* = 5, IFNα and IMQ topical and oral *n* = 3; *n* is the number of mice from one experiment. **h**, Lung metastasis was assessed by bioluminescence in 4T1 breast cancer-bearing mice from **g** 24 days after treatment start. **i**, Quantification of lung metastasis described in **h**. Control *n* = 5, IMQ topical + IFNα *n* = 4; *n* is the number of mice from one experiment. Data are plotted as mean ± s.e.m. Dots in **a**,**b** and **i** represent biological replicates. *P* values were calculated by one-way ANOVA with Tukey’s post-test (**a**) or unpaired, two-tailed *t*-test (**i**) with Welch’s correction (**b**) or two-way ANOVA with Tukey’s post-test (**c**–**g**). Source data

**Extended Data Fig. 2:**
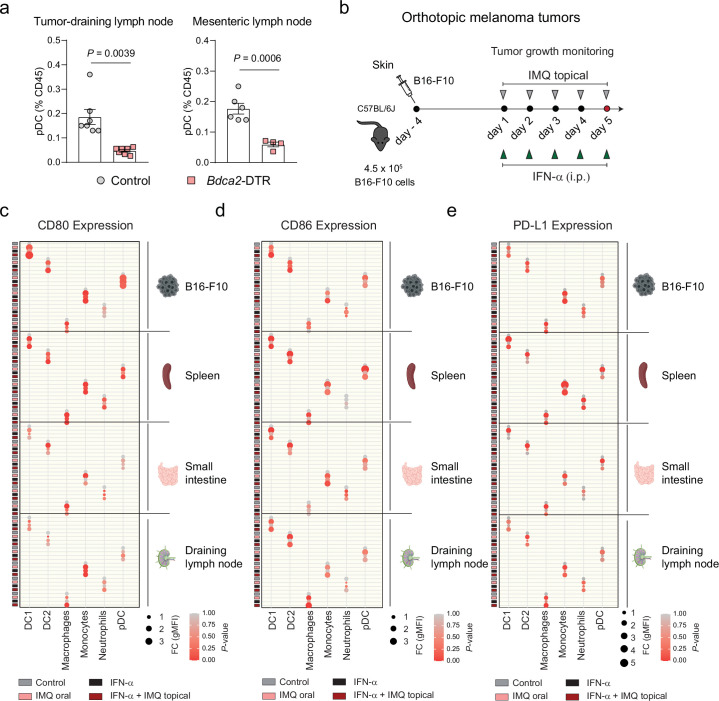
IFN-α induces maturation on myeloid cells across tissues. **a**, pDC depletion in *Bdca2*-DTR mice treated with Diphtheria toxin (DT) for 5 consecutive days. Mice were administered DT (4.5 ng DT/g, i.p.) every day, starting one day prior to IMQ treatment. pDCs are shown as the percentage among immune cells (CD45^+^ cells) in tumor-draining (td-LN) and mesenteric lymph nodes (m-LN). (Control: *n* = 7 td-LN, *n* = 6 m-LN; *Bdca2*-DTR: *n* = 7 td-LN, *n* = 4 m-LN; *n* is the number of mice pooled from 2 independent experiments). **b**, Schematic representation of the murine orthotopic melanoma model employed for IFN-α treatment: 4.5 ×10^5^ B16-F10 tumor cells were subcutaneously injected. IMQ treatment topical was done for five consecutive days in combination with recombinant IFN-α (10.000 U, i.p.). **c**, Expression of CD80 on myeloid cells across tissues. B16-F10 tumor-bearing mice were treated as described in (Fig. 3c). Dot plot shows the fold change (dot size) in the geometric mean fluorescence intensity (gMFI) on myeloid cells of treated mice to the control. Dot color indicates statistical significance, calculated using two-way ANOVA. In c-e *n* = 3 mice per group from 1 experiment. **d**, Expression of CD86 on myeloid cells across tissues as described in (c). **e**, Expression of PD-L1 on myeloid cells across tissues as described in (c). Bar graphs are plotted as mean ± SEM. Dots in (a) represent biological replicates. *P* values were calculated using unpaired, two-tailed *t*-tests with Welch´s correction (a). Dot plot graphs are plotted as mean (dot size) + *P* values (dot color). Source data

TLR7/8 are strongly expressed on pDCs and their activation leads to rapid production of type I IFNs (IFNα and IFNβ)^6^. We tested whether different administration routes of IMQ (topical, oral, combined topical and oral) differ in their ability to activate pDCs. We monitored type I IFN levels in plasma (IFN-I) 1 h after IMQ treatment and found that IFNα and IFNβ plasma levels increased exclusively in treatment groups receiving oral IMQ but remained low in the group treated only topically with IMQ (Fig. 2a). Notably, there was no induction of IFN-I in mice selectively depleted of pDCs (*Bdca2*-DTR mice) following oral IMQ treatment (Fig. 2b and Extended Data Fig. 2a) demonstrating that type I IFN production was strictly pDC dependent. Furthermore, IMQ treatment failed to control tumor growth in pDC-depleted mice (Fig. 2c) and in mice lacking the type I IFN receptor (*Ifnar1*^−/−^) (Fig. 2d)^17^. These results demonstrate that oral IMQ leads to IFN-I production by pDCs, which is essential for the antitumor effect of IMQ.

We next investigated whether systemic IFN-I could serve as a substitute for pDCs, and their activation by oral IMQ, as the latter can lead to systemic adverse events in patients^18^. Systemic IFNα administration restored the antitumor effect of oral and topical IMQ in mice depleted of pDCs, whereas IFNα treatment alone was not sufficient to replace IMQ in pDC-depleted melanoma-bearing mice (Fig. 2e and Extended Data Fig. 2b), suggesting that topical IMQ treatment was necessary in addition to systemic IFNα. Consistently, in wild-type mice, systemic treatment with IFNα alone failed to control tumor growth in the absence of IMQ, whereas the antitumor effect could be restored completely when systemic IFNα was combined with topical IMQ (Fig. 2f). Also, in the 4T1 breast cancer model the combination of systemic IFNα with topical IMQ inhibited the growth of primary tumors (Fig. 2g) and protected mice from the development of lung metastases (Fig. 2h,i). These results show that the oral TLR7/8 agonist can be substituted with systemic IFNα to achieve the full antitumor effect.

To further understand the impact of oral IMQ or systemic IFNα (with or without topical IMQ) on the antitumor immune response, we analyzed the expression of co-stimulatory/inhibitory molecules (CD80, CD86 and PD-L1) on myeloid cells in the tumor microenvironment, draining lymph node, spleen and small intestine. We observed that the myeloid immune cell compartment in different tissues showed a similar response to oral IMQ or systemic IFNα, regarding immune cell maturation, and consequently the ability to induce the adaptive immune system (for example, T cells) (Extended Data Fig. 2c–e).

Taken together, these results show that oral IMQ systemically activates pDCs to produce type I IFNs that are essential for the observed antitumor effect of IMQ at the treated (accessible) tumor site.

### IFNα sensitizes DCs and macrophages to TLR7/8 agonists

**Extended Data Fig. 3:**
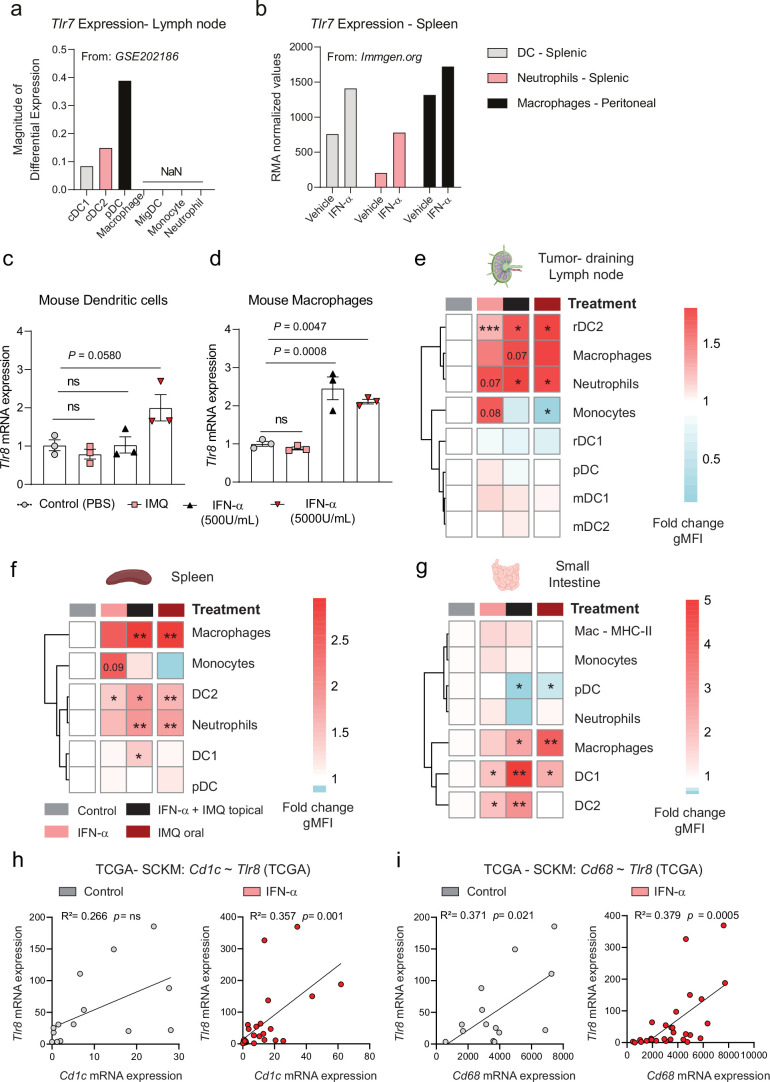
IFN-α therapy induces TLR7/8 expression on myeloid cells. **a**, Differential expression of *Tlr7* in myeloid cells identified by scRNA-Seq in skin-draining lymph nodes after IFN-α treatment (10.000 U, 4 h, s.c.) of mice. Gene expression data extracted from https://www.immunedictionary.org/app/home. **b**, *Tlr7* expression in sorted myeloid cells analyzed by Microarray. Gene expression data extracted from: http://rstats.immgen.org/Skyline_microarray/skyline.html?datagroup=IFN. IFN-α treatment: (10.000 U, 2 h). **c**, DCs were generated from bone marrow that was supplemented with FLT3L and *Tlr8* mRNA expression was analyzed using qRT–PCR. Cells were treated for 24 h with IFN-α (500 or 5000 U/mL), or with IMQ (2.5 µg/mL). In c, d *n* = 3 mice per group from 1 experiment. **d**, *Tlr8* mRNA expression was assessed in murine bone-marrow-derived macrophages using qRT–PCR. Macrophages were treated as described in (c). **e**, Heatmap depicts TLR7 expression on myeloid cells within tumor-draining lymph nodes. Treatments are indicated in color-coded rectangles above the heatmap. The fold change of the geometric mean fluorescence intensity (gMFI) of TLR7 on indicated myeloid cells of treated mice compared to the control is shown. In e–g *n* = 3 mice per group from 1 experiment. **f**, Spleens were analyzed for TLR7 expression. Heatmap was plotted as described in (e). **g**, Small intestines were analyzed for TLR7 expression. Heatmap was plotted as described in (e). **h**, *Tlr8* mRNA expression plotted against a human myeloid cell signature gene (*Cd1c*) in a subset of melanoma patients included in the TCGA database. The subset consisted of patients who received IFN-α prior to biopsy (*n* = 29) and a control group who did not receive IFN-α (*n* = 14). **i**, Correlation plot of *Tlr8* to *Cd68* in a subset of melanoma patients from the TCGA database, as described in (h). Bar graphs are plotted as mean ± SEM. Dots in c and d represent biological replicates. Correlation is shown in a xy-plot with a linear regression. Heatmaps are color-coded (blue = low, red = high value). *P* values were calculated using unpaired, two-tailed *t*-tests (e–g), one-way ANOVA with Tukey´s post-test (c, d) and two-tailed Pearson´s correlation test (h, i). * *P* < 0.05, ** *P* < 0.01, *** *P* < 0.001 and **** *P* < 0.0001. Source data

Next, we proceeded to investigate the underlying mechanism by which topical TLR7/8 agonists synergize with oral IMQ (or systemic IFNα) to achieve the antitumor effect. We hypothesized that IFNα could upregulate TLR7/8 on cells found in the microenvironment of the locally treated (accessible) tumors. We focused on myeloid cells, as we had shown previously that cells of the adaptive immune system were dispensable for the antitumor immune effect of IMQ occurring in the first week of treatment^7^. An in silico analysis of two available datasets showed that TLR7 expression was indeed upregulated on DCs in response to the IFNα cytokine (Extended Data Fig. 3a,b).

**Fig. 3:**
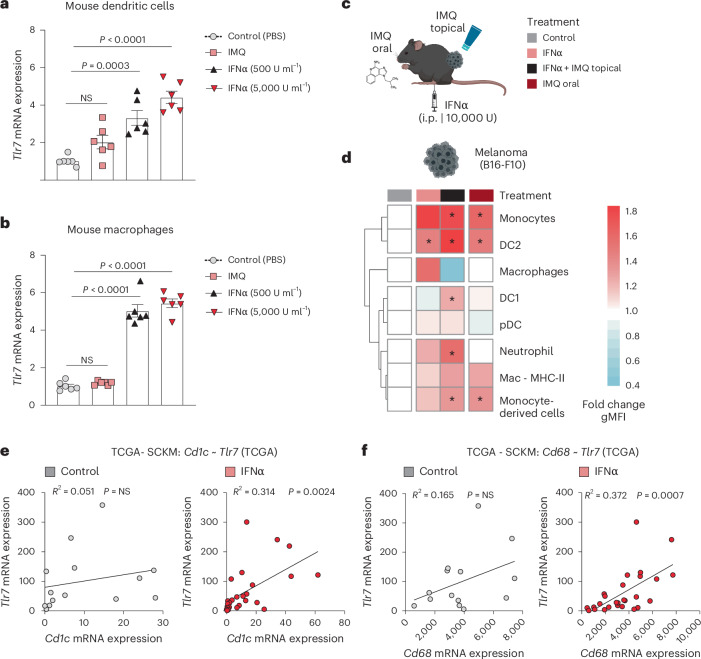
IFNα sensitizes DCs and macrophages to TLR7/8 agonists. **a**, Dendritic cells were generated from BM that was supplemented with FLT3L. Dendritic cells were treated with IFNα (500 or 5,000 U ml^−1^) or stimulated with IMQ (2.5 µg ml^−1^) for 24 h. *Tlr7* mRNA expression was analyzed using RT–qPCR. (*n* = 6 mice per group; data are pooled from two independent experiments). **b**, *Tlr7* mRNA expression was quantified in murine BM-derived macrophages treated as described in **a**. *n* = 6 mice per group; data are pooled from two independent experiments. **c**, Mice received IFNα and/or topical IMQ or oral IMQ 1 day before the analysis of TLR7 expression on myeloid cells by intracellular flow cytometry. Sample size: *n* = 3 mice per group from one experiment. **d**, Heatmap depicts TLR7 expression on myeloid cells within B16-F10 tumors. Treatments are indicated in color-coded rectangles above the heatmap, as described in **c**. The fold change of the geometric mean fluorescence intensity (gMFI) of TLR7 on myeloid cells of treated mice compared to the control is shown. **e**, *Tlr7* mRNA expression was plotted against a human dendritic cell signature gene (*Cd1c*) in a subset of patients with melanoma included in the TCGA database. The subset consisted of patients who received IFNα before biopsy (*n* = 29) and a control group of patients who did not (*n* = 14). **f**, Correlation plot of *Tlr7* to *Cd68*, a human macrophage cell signature gene, in a subset of melanoma patients from the TCGA database described in **e**. Bar graphs are plotted as mean ± s.e.m. Dots in **a** and **b** represent biological replicates. Correlation is shown in a *xy*-plot with a linear regression. Heatmaps are color-coded (blue, low; red, high value). *P* values were calculated by unpaired, two-tailed *t*-tests (**d**), one-way ANOVA with Tukey’s post-test (**a**,**b**) and two-tailed Pearson’s correlation test (**e**,**f**). Source data

In vitro, we found that mouse bone-marrow-derived DCs and macrophages both upregulate *Tlr7* and *Tlr8* messenger RNA levels, when incubated with IFNα, but not when incubated with IMQ (Fig. 3a,b and Extended Data Fig. 3c,d). In vivo, IFNα induced TLR7 protein expression in myeloid cells across different tissues (Fig. 3c,d and Extended Data Fig. 3e–g). Notably, a single-dose of IFNα was sufficient to upregulate TLR7 protein expression on type II DCs and the combination of IFNα and topical IMQ-induced TLR7 expression also on type I DCs in the tumor microenvironment (Fig. 3c,d). These findings show that IFNα enhances TLR7 expression on myeloid cells.

To demonstrate the human relevance of our findings, we conducted a comparative analysis of *Tlr7/8* mRNA expression and its correlation with signature genes of various cell types in tumor biopsies of patients who received type I IFN (*n* = 29) and who did not (*n* = 14) (The Cancer Genome Atlas (TCGA)–Skin Cutaneous Melanoma project (SKCM)). As expected, in both patient groups *Tlr7/8* mRNA expression correlated with immune cells genes (Supplementary Table 1). Other cell types, such as epithelial cells did not correlate with TLR7/8 in patients; however, in patients, who received IFNα therapy, we observed a significant positive correlation between *Tlr7/8* expression and signature genes associated with myeloid cells, such as *Cd1c* and *Cd68* (Fig. 3e,f and Extended Data Fig. 3h,i). These findings suggest that IFNα increases *Tlr7/8* mRNA expression on myeloid immune cells, but not on other stromal cells, such as epithelial cells.

**Fig. 4:**
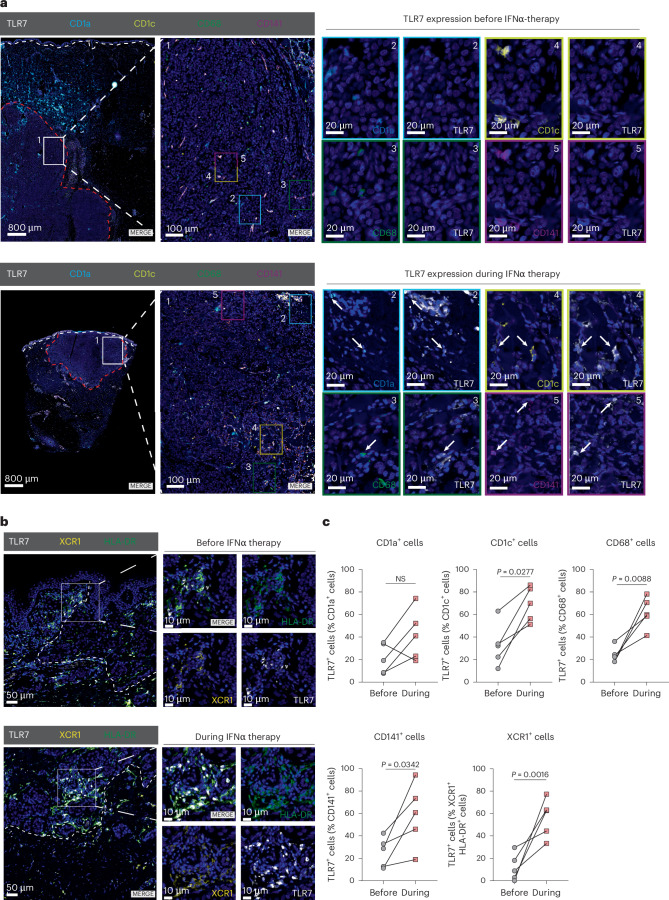
IFNα therapy induces TLR7 expression on myeloid cells. **a**, Multiplex immunofluorescence analysis of melanoma samples from a cohort of *n* = 5 patients who underwent IFNα therapy. The staining panel included antibodies against CD1a, CD1c, CD68, CD141 and TLR7. Representative images are shown for a patient before (top) and during IFNα therapy (bottom). The white-dotted line demarcates the border of the epidermis and the red-dotted line indicates the border of the tumor. Arrows indicate double-positive cells, which are shown magnified in numeric and color-coded insets. Magnification: ×0.65, ×4.5 and ×35 (zoom). Scale bars, 800 µm, 100 µm and 20 µm. **b**, Immunofluorescence staining against TLR7, XCR1 and HLA-DR in a cohort of patients with melanoma described in **a**. Representative images are shown. The inset magnifies an area of interest. The white-dotted line shows tumor border. Scale bars, 50 µm and 10 µm. **c**, Quantification of TLR7-expressing cells positive for CD1a, CD1c, CD68 and CD141 (**a**) or double-positive for XCR1 and HLA-DR (**b**) in tumor parenchyma was performed in melanoma patients as described in **a** (*n* = 5 individuals). Data are plotted as dots and lines. Dots in **c** represent five individuals. *P* values were calculated using paired, two-tailed *t*-tests (**c**). Source data

**Extended Data Fig. 4:**
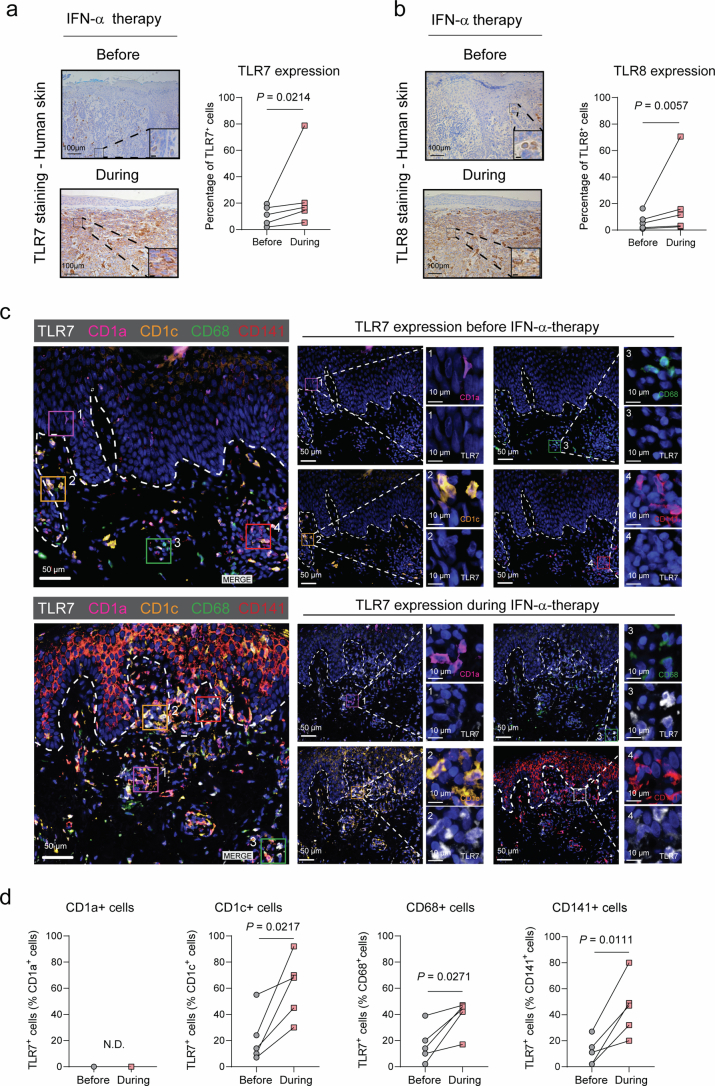
IFN-α therapy induces TLR7 on myeloid cells in tumor stroma. **a**, TLR7 immunohistochemistry staining of human skin. To the left: Representative pictures before and during IFN-α therapy. Insets show enlarged views of the marked areas. To the right: Quantification of TLR7-positive cells is presented as a percentage of total cells. Scale bar: 100 µm. In a, b *n* = 5 individuals. **b**, TLR8 staining of human skin of patients undergoing IFN-I therapy. On the left are representative pictures. Insets provide enlarged views of the marked areas. On the right is the quantification of TLR8-positive cells which is shown as percentage of total cells. Scale bar: 100 µm. **c**, Multiplex immunofluorescence analysis of melanoma samples from a cohort of five patients undergoing IFN-α therapy. The staining panel included antibodies against CD1a, CD1c, CD68, CD141, and TLR7. Representative images show the upper dermis of a patient before (upper panels) and during IFN-α therapy (lower panels). On the left, a full-color panel, and on the right TLR7 in combination with selected markers is shown. Insets show enlarged views of the marked areas, and highlight cells that are positive for TLR7 and/or one of the myeloid cell markers. Magnification: 10x (Zoom), Scale bar: 50 µm, Insets: 10 µm. In d *n* = 5 individuals. **d**, Quantification of TLR7-expressing cells positive for CD1a, CD1c, CD68 and CD141 was performed in the upper dermis in immunofluorescence staining’s described in (c). N.D. = not detectable. Data are plotted as dots and lines. Dots in a, b and represent individuals. *P* values were calculated using paired, two-tailed *t*-test (a, b and d). Source data

To confirm our in silico prediction, we performed immunohistochemistry (Extended Data Fig. 4a,b) and multiplex immunofluorescence for TLR7/8 in an independent cohort of patients with cutaneous melanoma (Fig. 4a,b, Extended Data Fig. 4c and Supplementary Figs. 1 and 2). For every patient (*n* = 5), we had matched biopsies of skin tumors before and during IFNα treatment. Multiplex immunofluorescence analysis revealed that TLR7 was significantly upregulated by IFNα therapy in cells expressing myeloid cell markers such as CD1c^+^, CD68^+^ and CD141^+^ and in XCR1^+^ cells, a marker specific for type I DCs (Fig. 4a–c) in the tumor and the surrounding stroma (Extended Data Fig. 4c,d). Notably, CD1a^+^ cells showed TLR7 expression only when they were infiltrating the tumor, but not in the stroma (Fig. 4c and Extended Data Fig. 4d). Taken together these results suggest that the synergy between oral and topical IMQ is based on type I IFN induction in pDCs by oral IMQ, followed by upregulation of TLR7/8 on myeloid cells such as DCs, possibly sensitizing these immune cells to topical TLR7/8 agonist treatment at tumor sites.

### TLR7-c-Jun signaling in DCs promotes IL-12 expression

**Extended Data Fig. 5:**
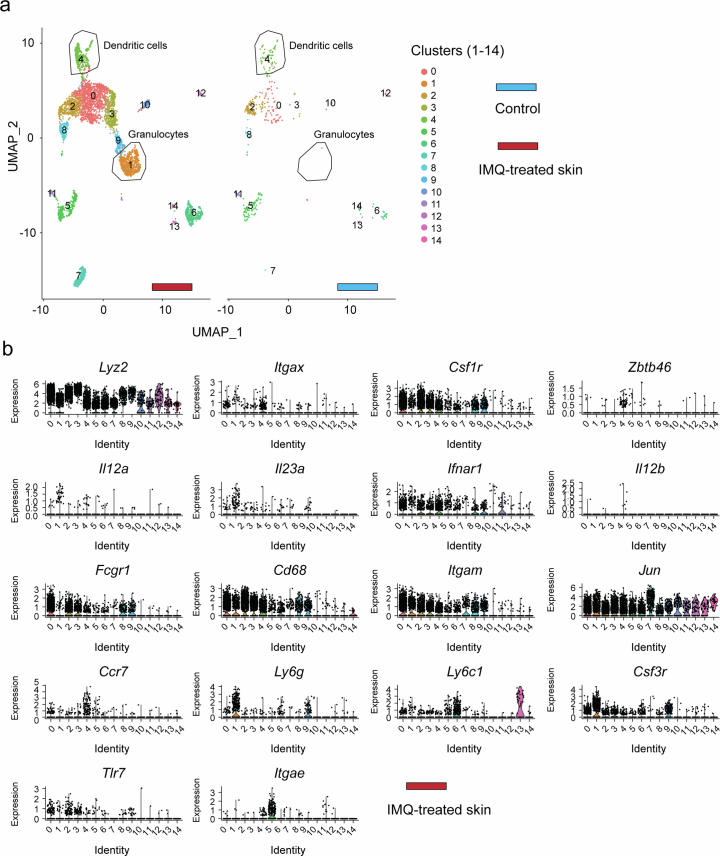
Dendritic cells are the primary source of IL-12 in IMQ-treated skin. Uniform Manifold Approximation and Projection (UMAP) generated from a previously published scRNA-seq dataset (GSE150361) comparing immune cells in IMQ-treated skin to untreated control. Violin plots show the expression levels of indicated mRNA transcripts in the scRNA-Seq dataset described in (**a**). Signature genes that are associated with DCs (for example, *Zbtb46* and *Itgax*) or macrophages (for example, *Fcgr1*, *Cd68*) are displayed in (**b**).

We next explored the molecular pathways and effectors blocking tumor growth in locally treated (accessible) sites. Given the profound IFNα-driven TLR7/8 upregulation on DCs and macrophages, we searched for factors produced by these cells in response to IMQ. For this purpose, a published single-cell RNA sequencing (scRNA-seq) dataset of IMQ-treated skin was reanalyzed^19^. Among all the factors identified (Extended Data Fig. 5a,b), we focused on the cytokine IL-12, known for its potent antitumorigenic properties^20^. IL-12 expression was detectable in myeloid cells, with the *Il-12a* subunit being prominently expressed in cluster 1 (granulocytes) and cluster 4 (DCs) and the *Il-12b* subunit being almost exclusively expressed in cluster 4. Moreover, previous studies showed that DCs and macrophages can produce IL-12 in response to TLR7/8 agonists^21,22^.

**Fig. 5:**
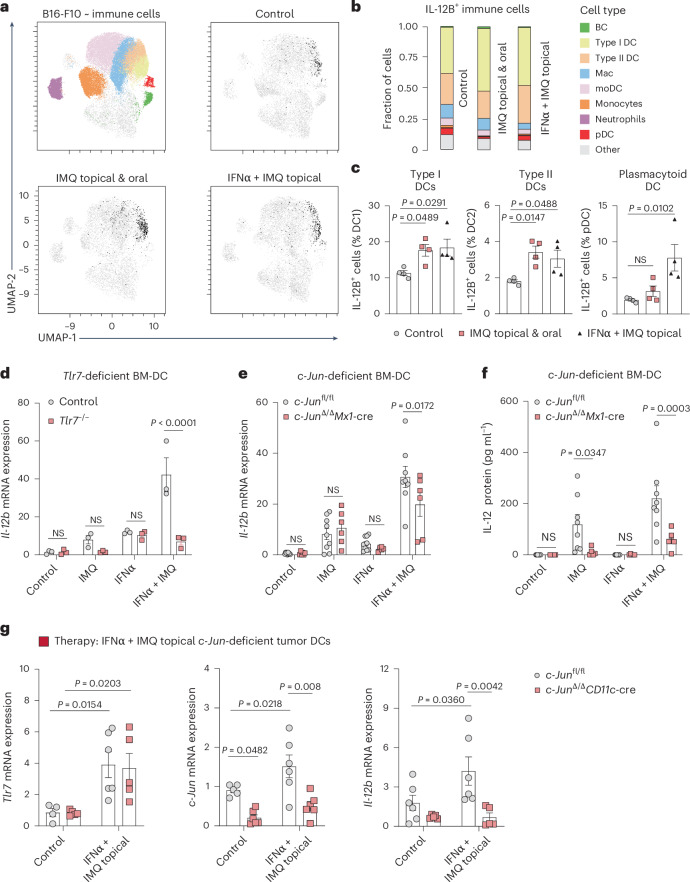
TLR7-c-Jun signaling in DCs promotes IL-12 expression. **a**–**c**, UMAP plot of immune cells (CD45^+^) in B16-F10 tumors shows the distribution of IL-12B in the myeloid cell compartment. Treatment is indicated. Identified cell clusters are color-coded (top right). Fraction of cells positive for IL-12B (**b**). Cells are assigned to cell clusters (color) identified in UMAP plot. Percentage of type I or II DCs and pDCs positive for IL-12B (**c**). Classical gating (*n* *=* 6 mice per group; data are pooled from two independent experiments). **d**, *Il-12b* mRNA expression was quantified in murine BM-DCs generated from wild-type (control) and *Tlr*7^−/−^ BM. BM-DCs were either pre-treated with IFNα (500 U ml^−1^) for 6 h and/or stimulated with IMQ (2.5 µg ml^−1^) over night. (*n* = 3 mice per group from one experiment). **e**, *Il-12b* mRNA expression was quantified by RT–qPCR in BM-DCs generated from *c-Jun*^fl/fl^ and *c-Jun*^∆/∆^*Mx1*-Cre BM. Treatments are as described in **d**. (*c-Jun*^fl/fl^: control *n* = 9, IMQ *n* = 9, IFNα *n* = 9, IFNα + IMQ *n* = 8, *c-Jun*^∆/∆^*Mx1*-Cre *n* = 6 mice per group; data are pooled from three independent experiments). **f**, IL-12 protein levels were determined by ELISA in the supernatants of BM-DCs from mice of indicated genotype and treated as described in **d**. *c-Jun*^fl/fl^ *n* = 8 mice per group, *c-Jun*^∆/∆^*Mx1*-Cre *n* = 6 mice per group; data are pooled from two independent experiments. **g**, *Tlr7*, *c-Jun*, and *Il-12b* mRNA expression was analyzed by RT–qPCR in sorted DCs (CD45^+^CD64^-^CD11c^+^MHC-II^+^) isolated from B16-F10 tumors implanted in *c-Jun*^fl/fl^ and *c-Jun*^∆/∆^*CD11c*-Cre mice. Tumors were topically treated with IMQ and the mice received IFNα. *Tlr7* mRNA: *c-Jun*^fl/fl^ *n* = 4 or IFNα + IMQ topical *n* = 6 mice and *c-Jun*^*Δ/Δ*^*CD11c*-Cre *n* = 6 mice per group, *c-Jun* mRNA: *n* = 6 mice per group or *c-Jun*^fl/fl^ control *n* = 5 mice, *Il-12b* mRNA: *c-Jun*^fl/fl^ *n* = 6 mice and *c-Jun*^*Δ/Δ*^*CD11c*-Cre *n* = 5 mice per group; data are pooled from two independent experiments. Data are plotted as mean ± s.e.m. Dots in **c**–**g** represent biological replicates. *P* values were calculated using one-way ANOVA with Dunnett’s post-test (**c**) and two-way ANOVA with Sidak’s post-test (**d**–**g**). Source data

**Extended Data Fig. 6:**
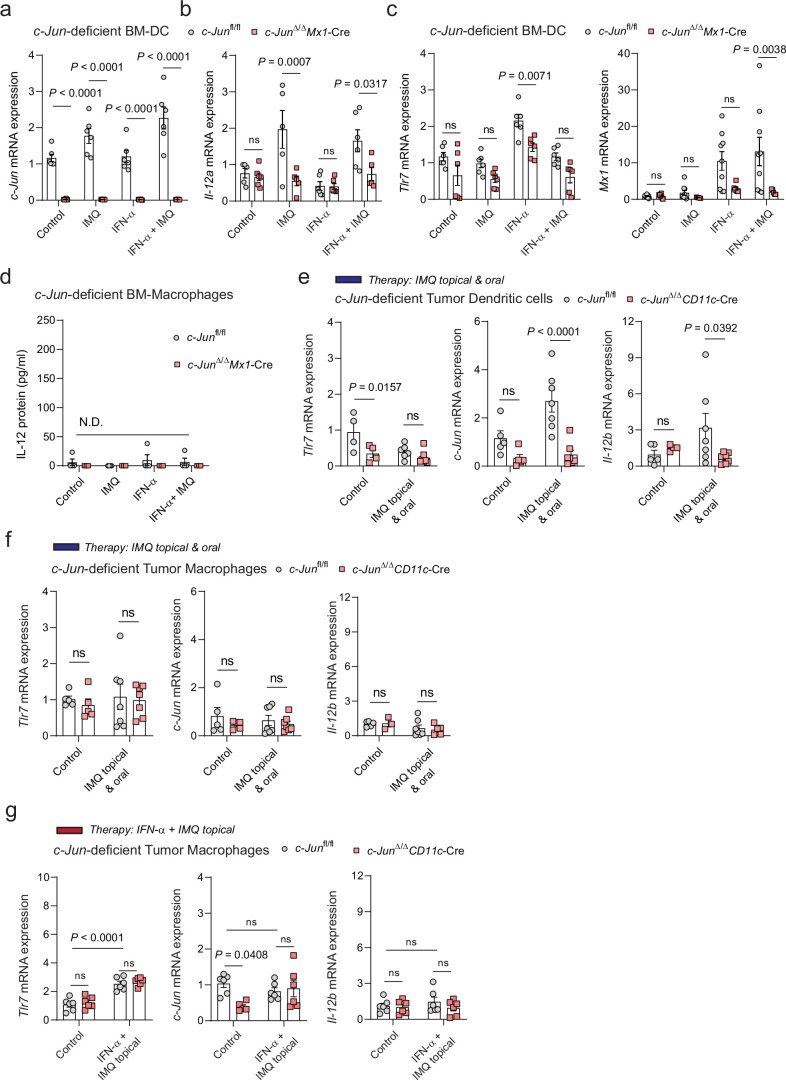
c-Jun modulates the immune response in DCs and macrophages downstream of TLR7. **a**, *c-Jun* mRNA expression levels were measured in murine bone-marrow-derived DCs using qRT–PCR. Cells were pre-treated with IFN-α (6 h) and/or stimulated with IMQ over night. (*n* = 6 mice per group; Data are pooled from 2 independent experiments). **b**, *Il-12a* mRNA expression levels were assessed by qRT–PCR in BM-DCs as described in (a). (*n* = 6 mice, *c-Jun*^*fl/fl*^*:* Control *n* = 5, IMQ *n* = 5, *c-Jun*^*Δ/Δ*^*Mx1-Cre:* IMQ *n* = 5, IFN-α + IMQ *n* = 5; *n* is the number of mice pooled from 2 independent experiments). **c**, *Tlr7* and *Mx1* mRNA expression levels were assessed by qRT–PCR in BM-DCs as described in (a). **d**, IL-12 protein levels were measured by ELISA in the supernatant of bone-marrow-derived macrophages following the indicated treatment. N.D.: Not detectable; (*c-Jun*^*fl/fl*^ *n* = 4 mice, *c-Jun*^*Δ/Δ*^*Mx1-Cre*; *n* = 3 mice; 1 experiment). **e**, *Tlr7*, *c-Jun*, and *Il-12b* mRNA expression levels were analyzed by qRT–PCR in tumor-infiltrating DCs (CD45^+^CD64^-^CD11c^+^MHC-II^+^) that were isolated from B16-F10 tumors of mice with the indicated genotype and treated with IMQ topically and orally. *Tlr7*: Control group *n* = 5, IMQ topical &oral: *c-Jun*^fl/fl^ *n* = 6, *c-Jun*^Δ/Δ^*Mx1*-Cre *n* = 7, *c-Jun:* Control group *n* = 5, IMQ topical &oral group *n* = 7, *Il12-b*: Control group: *c-Jun*^fl/fl^ *n* = 5, *c-Jun*^Δ/Δ^*Mx1*-Cre *n* = 4, IMQ topical &oral group *n* = 7; *n* is the number of mice pooled from 2 independent experiments. **f**, *Tlr7*, *c-Jun*, and *Il-12b* mRNA expression levels were analyzed by qRT–PCR in tumor-associated macrophages (CD45^+^CD64^+^CD11b^+^), as described in (e). *Tlr7* and *c-Jun*: Control group *n* = 5, IMQ topical &oral *n* = 7, *Il-12b*: Control group: *c-Jun*^fl/fl^ *n* = 5, *c-Jun*^Δ/Δ^*CD11c*-Cre *n* = 3, IMQ topical &oral: *c-Jun*^fl/fl^ *n* = 7, *c-Jun*^Δ/Δ^*CD11c*-Cre *n* = 5; *n* is the number of mice pooled from 2 independent experiments. **g**, *Tlr7*, *c-Jun*, and *Il-12b* mRNA expression levels were analyzed by qRT–PCR in sorted macrophages (CD45^+^CD64^+^CD11b^+^). Macrophages were isolated from B16-F10 tumors that were implanted in *c-Jun*^fl/fl^ and *c-Jun*^∆/∆^*CD11c*-Cre mice. Tumors were treated with IMQ topically and mice received IFN-α (see Extended Data Fig. 2b). *Tlr7 n* = 6 mice per group, *c-Jun* and *Il-12b*: *n* = 6 mice per group and in the Control: *c-Jun*^Δ/Δ^*CD11c*-Cre group *n* = 5 mice; n is the number of mice pooled from 2 independent experiments. Data are plotted as mean ± SEM. Dots in a-g represent biological replicates. *P*‐values were calculated using two-way ANOVA with Sidak´s post-test. (a-g). Source data

To further define the cellular source of IL-12 in the TME in vivo, we performed an intracellular flow cytometry staining of IL-12B in myeloid cells derived from tumors of treated mice (Fig. 5a). Both type I (~50%) and type II DCs (~20%) produced the majority of IL-12B in response to one-time combination therapy (topical IMQ and systemic IFNα or oral IMQ). Other myeloid cells, such as macrophages (10%), only showed a minor contribution to IL-12B production (Fig. 5b,c). Thus, DCs were analyzed further with the aim to investigate the molecular mechanism of IL-12 induction downstream of TLR7/8 signaling. In bone-marrow-derived DCs we noticed that the expression of *Il-12b* mRNA was strongly induced in response to IMQ, when cells were pre-incubated with IFNα, whereas *Il-12b* mRNA was only weakly induced by either IMQ or IFNα treatment alone (Fig. 5d). DCs derived from mice lacking TLR7 (*Tlr7*^−/−^)^3^ failed to do so (Fig. 5d), confirming that IL-12 production is mediated by TLR7 signaling.

c-Jun/AP-1 plays a critical role in DCs downstream of TLR7 (ref. ^10^) and we, therefore, hypothesized that c-Jun could be a relevant factor involved in IL-12 expression. Indeed, we found that c-Jun-deficient DCs, generated from the bone marrow of *c-Jun*^∆/∆^*Mx1*-Cre mice (Extended Data Fig. 6a), had reduced *Il-12a* and *Il-12b* mRNA expression in vitro after treatment compared to control mice (Fig. 5e and Extended Data Fig. 6b). Of note, IL-12 protein was also reduced in the supernatant of c-Jun-deficient BM-DCs (Fig. 5f). Lack of *c-Jun* in BM-DCs reduced the expression of the IFN-I-induced gene *Mx1*, but did not directly affect *Tlr7* expression after combinatorial treatment (Extended Data Fig. 6c). Notably, we could not detect significant IL-12 protein expression in macrophages in vitro, confirming DCs as the major source of IL-12 (Extended Data Fig. 6d).

Next, we investigated DCs in vivo that were sorted from melanoma-bearing mice lacking *c-Jun* exclusively in DCs (*c-Jun*^Δ/Δ^*CD11c-*Cre) and that were treated with IMQ orally and topically. We observed that *c-Jun* was induced in DCs upon treatment and critical for the induction of *Il-12b* in DCs, but not in macrophages (Extended Data Fig. 6e,f).

To further demonstrate that systemic IFNα is a potent substitute for oral IMQ in vivo, we also sorted DCs from tumors implanted in *c-Jun*^Δ/Δ^*CD11c-*Cre mice treated with IMQ topically and IFNα systemically. Consistent to the in vitro data, *Tlr7* mRNA expression was upregulated and not affected by the lack of c-Jun in DCs (Fig. 5g). *Il-12b* mRNA expression was also induced by combined IFNα and IMQ treatment and reduced in DCs lacking *c-Jun* (Fig. 5g). In contrast to DCs, sorted macrophages showed only a weak induction of *Tlr7* mRNA expression in response to the combinatorial treatment (IFNα and IMQ) and the expression of *Il-12b* mRNA was unchanged (Extended Data Fig. 6g). These results demonstrate that IFN-I-primed DCs require the transcription factor c-Jun/AP-1 to efficiently produce IL-12 after IMQ treatment.

### The IMQ antitumor effect depends on c-Jun signaling in DCs

**Fig. 6:**
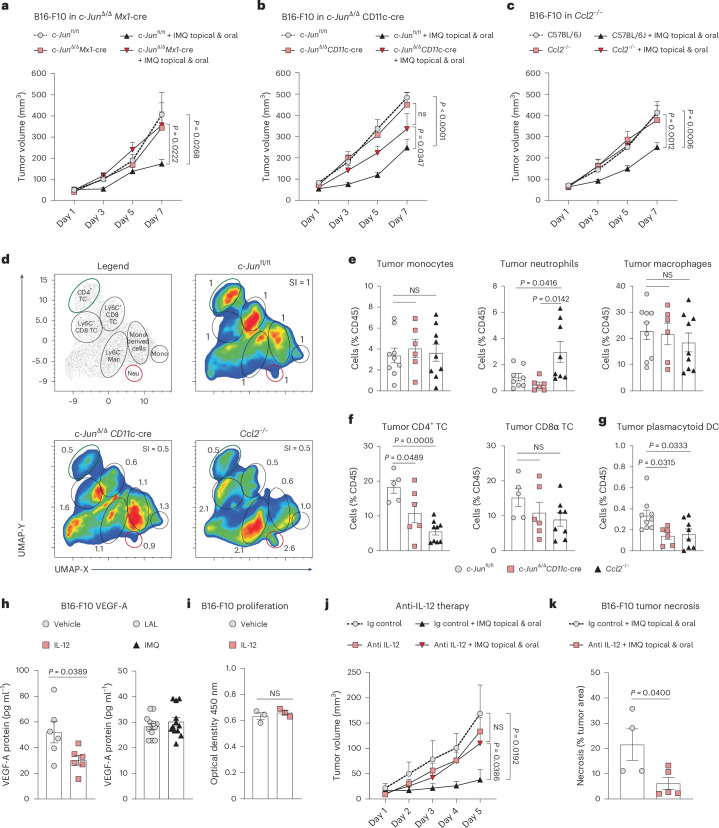
The IMQ antitumor effect depends on c-Jun signaling in DCs. **a**–**c**, Tumor growth was monitored in *c-Jun*^fl/fl^ and *c-Jun*^Δ/Δ^*Mx1*-Cre mice (*c-Jun*^fl/f^: *n* = 9, IMQ topical and oral *n* = 9, *c-Jun*^Δ/Δ^*Mx1*-Cre: *n* = 4, IMQ topical and oral *n* = 8) (**a**), in *c-Jun*^fl/fl^ and *c-Jun*^Δ/Δ^*CD11c*-Cre (*c-Jun*^fl/f^: *n* = 19, IMQ topical and oral *n* = 18, *c-Jun*^Δ/Δ^*CD11c*-Cre: *n* = 19, IMQ topical and oral *n* = 17) (**b**) and wild-type and *Ccl2*^−/−^ mice (C57BL/6J: *n* = 18, IMQ topical and oral *n* = 20, *Ccl2*^−/−^: *n* = 13, IMQ topical and oral *n* = 19) (**c**). *n* in **a**–**c** is the number of mice pooled from two (**a**), four (**b**) and five (**c**) independent experiments. **d**, UMAP analysis was performed on tumor-infiltrating immune cells obtained from mice of indicated genotype, 1 day after IMQ therapy ended. Clustering with CD4, CD8, CD11b, CD64, Ly6-C, Ly6-G and TCRβ. The legend plot shows the identified populations. The fold change of each cluster to the control (*c-Jun*^fl/fl^) is depicted. *n* = 3 mice per group from one experiment. **e**–**g**, Frequency of monocytes, neutrophils and macrophages (**e**), CD4^+^ and CD8^+^ T cells (**f**) and pDCs (**g**) among tumor-infiltrating immune cells in *c-Jun*^fl/fl^, *c-Jun*^Δ/Δ^*CD11c*-Cre and *Ccl2*^−^^/−^ mice 1 d after IMQ therapy ended. *c-Jun*^fl/fl^: *n* = 9 (monocytes and macrophages), *n* = 8 (neutrophils) (**e**), *n* = 5 (**f**), *n* = 9 (**g**), *c-Jun*^*Δ/Δ*^*CD11c*-Cre: *n* = 8 (**e**–**g**), *Ccl2*^−/^^−^: *n* = 9 (monocytes and macrophages), *n* = 8 (neutrophils) (**e**), *n* = 9 (CD4^+^ T cells), *n* = 8 (CD8α T cells) (**f**), *n* = 8 (**g**); *n* in **e**–**g** is the number of mice pooled from two (**e**,**f**) or three (**g**) independent experiments. **h**, Protein levels of VEGF-A were measured by ELISA in B16-F10 cells treated with IL-12B (10 ng ml^−1^) or IMQ (2.5 µg ml^−1^) for 24 h. IL-12B: *n* = 6 and IMQ: *n* = 12 technical replicates of B16-F10 cells per group pooled from two independent experiments. **i**, B16-F10 proliferation after treatment with IL-12B as described in **h**. *n* = 3 technical replicates of B16-F10 cells per group from one experiment. **j**, Tumor growth kinetics in wild-type mice implanted with B16-F10 melanoma cells. The mice received combination therapy and/or anti-IL-12 antibody (500 µg day^−1^). *n* = 4 mice per group or Ig control + IMQ topical and oral *n* = 6 mice; data are pooled from two independent experiments. **k**, Quantification of the necrotic area in tumors treated as described in **j**. Ig control *n* = 4, anti-IL-12 *n* = 5; *n* is the number of tumors pooled from two independent experiments. Data are shown as mean ± s.e.m. Dots represent biological replicates (**e**–**g**) and technical replicates (**h**,**i**). *P* values were calculated using unpaired, two-tailed *t*-tests (**h**,**i**,**k**), one-way ANOVA (**e**–**g**) or two-way ANOVA both with Tukey’s post-test (**a**–**c**,**j**). Source data

To assess the role of c-Jun in myeloid cells for the antitumor activity of IMQ in vivo, we used *c-Jun*^Δ/Δ^*Mx1*-Cre mice and *c-Jun*^Δ/Δ^*CD11c-*Cre mice. Notably, in the *Mx1*-Cre mouse model deletion occurs in IFN-I responsive cells, which we have previously shown to mediate the antitumor effect of IMQ^7^. In addition, as c-Jun regulates the expression of the pDC-recruiting chemokine CCL2 in DCs^10^, we employed *Ccl2*^−/−^ mice. We found that the antitumor effect of IMQ was strongly diminished in *c-Jun*^Δ/Δ^*Mx1*-Cre mice (Fig. 6a), *c-Jun*^Δ/Δ^*CD11c-*Cre mice (Fig. 6b) and *Ccl2*^−/−^ mice (Fig. 6c); however, the lack of *c-Jun* or *Ccl2* did not have a significant impact on the growth of orthotopic B16-F10 melanomas in untreated control mice.

**Extended Data Fig. 7:**
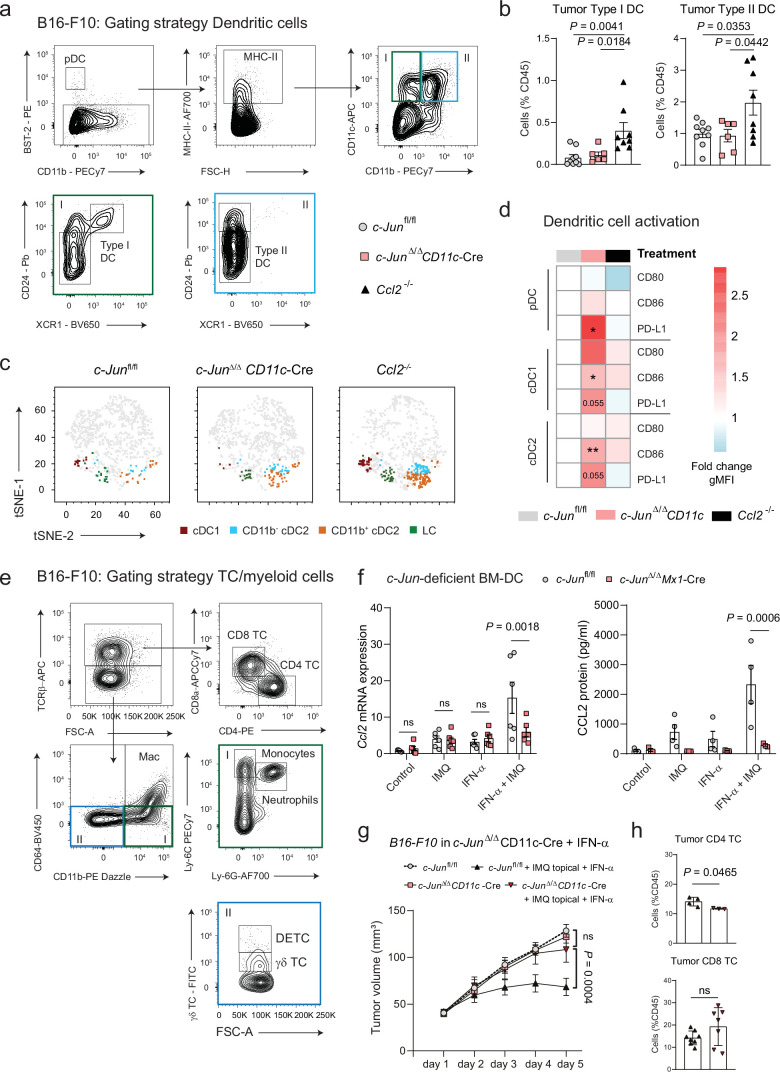
Impact of c-Jun-CCL2 signaling on the immune cell profile of IMQ-treated B16 tumors. **a**, Gating strategy to identify pDCs and Type I (CD11b^-^CD24^+^XCR1^+^) and Type II DCs (CD11b^+^CD24^-^XCR1^-^) in B16-F10 tumor-bearing mice at therapy end point (Day 8). **b**, Frequency of Type I and Type II DCs identified as defined in (a). (*c-Jun*^fl/fl^ *n* = 9, *c-Jun*^Δ/Δ^*CD11c*-Cre *n* = 6, *Ccl2*^−/−^ *n* = 8; *n* is the number of mice pooled from 3 independent experiments). **c**, *t*-SNE plots show the distribution of DC subpopulations in B16-F10 tumors from (a). *t*-SNE analysis was performed on CD45^+^CD64^-^MHC-II^+^ immune cells (*n* = 3 mice per group; 1000 cells) with the surface markers for CD11b, CD11c, CD24 and XCR1. **d**, Heatmap shows expression of the maturation markers CD80, CD86 and PD-L1 on DCs within B16-F10 tumors at end of combination therapy (Day 5). The fold change of the geometric mean fluorescence intensity is shown. *c-Jun*^Δ/Δ^*CD11c*-Cre or *Ccl2*^−/−^ DC activation markers are compared to the control (*c-Jun*^fl/fl^). (n *=* 3 mice per group from 1 experiment). *P *<* 0.05, ** *P* *<* 0.01 and *** *P* *<* 0.001. **e**, Gating strategy to identify T cells (TC; TCRβ^+^) positive for CD4 or CD8α, monocytes (CD11b^+^ Ly6-C^+^Ly6-G^-^), neutrophils (CD11b^+^ Ly6-C^int^Ly6-G^+^) and γδ T cells (TCRγδ^int^) in B16-F10 tumor-bearing mice on IMQ-therapy end point (Day 8). **f**, CCL2 expression levels were assessed in BM-DCs of the indicated genotype using qRT–PCR (left) and ELISA (right) assays. The cells were treated as described in Extended Data Fig. 6a. (Left: *n* = 6 mice per group from 3 independent experiments, Right: *c-Jun*^*fl/fl*^: *n* = 4 mice per group, *c-Jun*^*Δ/Δ*^*CD11c-Cre n* = 3 mice per group from 1 experiment). **g**, B16-F10 tumors were implanted in *c-Jun*^fl/fl^ and *c-Jun*^Δ/Δ^*CD11c*-Cre mice treated topically with IMQ, and systemically with IFN-α (see Extended Data Fig. 2b). (*c-Jun*^fl/f^: *n* = 6, IMQ topical + IFN-α *n* = 7, *c-Jun*^Δ/Δ^*CD11c*-Cre: *n* = 8, IMQ topical + IFN-α *n* = 8, *n* is the number of mice pooled from 2 independent experiments). **h**, Tumor-immune cell infiltrate was analyzed by flow cytometry in B16-F10 tumors from (g). IMQ topical + IFN-α: *c-Jun*^*fl/fl*^ *n* = 4 CD4, *n* = 8 CD8 TC; *c-Jun*^Δ/Δ^*CD11c*-Cre *n* = 3 CD4, *n* = 7 CD8 TC; *n* is the number of mice pooled from 2 independent experiments (CD8) or 1 experiment (CD4 TC). Data are plotted as mean ± SEM. Dots in b, f and h represent biological replicates. *P*‐values were calculated using unpaired, two-tailed *t*-tests with Welch´s correction (h), one-way ANOVA with Tukey´s post-test (b) and two-way ANOVA with Sidak´s post-test (d, f, g). Source data

Effective cancer immunotherapy relies on the infiltration of antitumorigenic immune cells, particularly CD8^+^ T cells. We, therefore, next characterized the composition of the tumor-immune infiltrate. We found that IMQ-treated tumors in *c-Jun*^Δ/Δ^*CD11c-*Cre and *Ccl2*^−/−^ mice had substantially reduced CD4^+^ T cells, but CD8^+^ T cells were unchanged (Fig. 6d–f). Additionally, in *c-Jun*^Δ/Δ^*CD11c-*Cre tumors, DCs and pDCs exhibited elevated expression of CD86 and PD-L1, whereas tumors from *Ccl2*^−/−^ mice showed an increase in neutrophils and DCs, with unaltered DC activation (Fig. 6e and Extended Data Fig. 7a–e). Consistent with previous findings of our group^7^, *Ccl2*^−/−^ mice showed reduced frequencies of tumor-infiltrating pDCs, confirming the critical role of CCL2 in pDC recruitment. Similarly, in *c-Jun*^Δ/Δ^*CD11c-*Cre mice, the infiltration of pDCs was diminished (Fig. 6g), in line with the observed reduced expression of CCL2 in *c-Jun*-deficient BM-DCs (Extended Data Fig. 7f). Moreover, the antitumor effect of IFNα–IMQ combination therapy was abolished in treated *c-Jun*^Δ/Δ^*CD11c-*Cre mice, with a reduction in tumor-infiltrating CD4^+^ T cells (Extended Data Fig. 7g,h).

**Extended Data Fig. 8:**
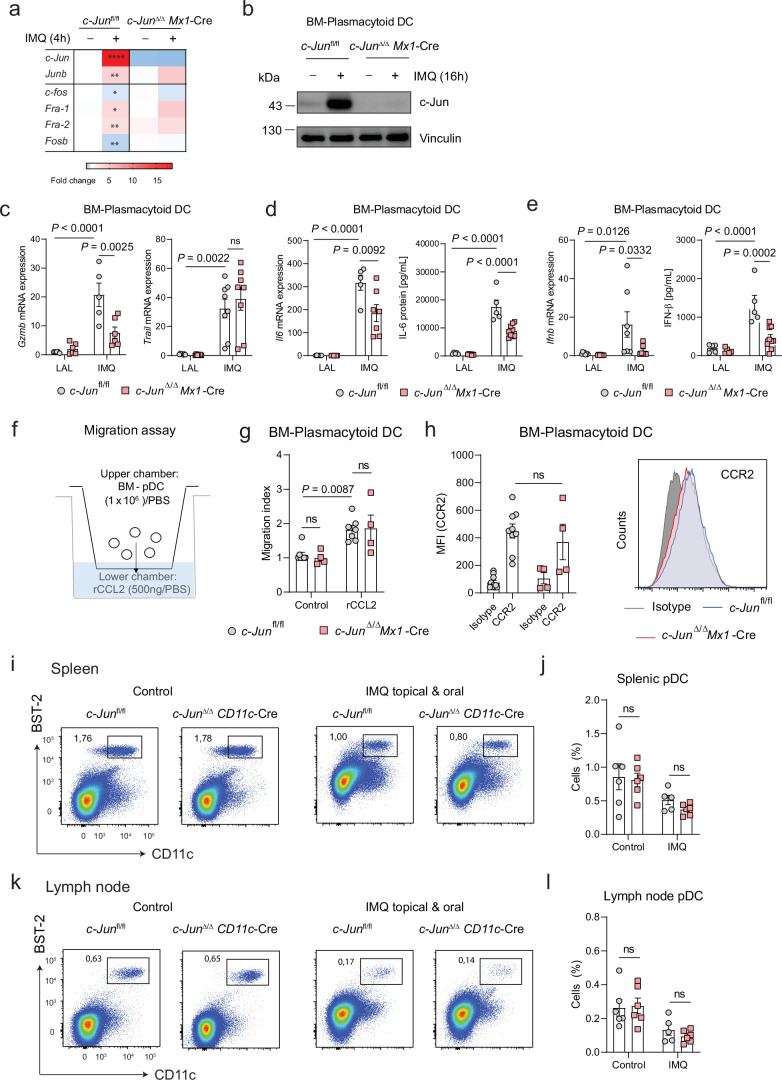
Role of c-Jun in the development and function of pDCs. **a**, Heatmap depicts AP-1 family member expression in *c-Jun*^fl/fl^ and *c-Jun*^Δ/Δ^*Mx1*-Cre bone-marrow-derived (BM) -pDCs stimulated with IMQ (4 h). The heatmap shows the fold change compared to LAL-treated *c-Jun*^fl/fl^ BM-pDCs as determined by qRT–PCR analysis. Asterisk shows significant differences in *c-Jun*^fl/fl^ IMQ versus *c-Jun*^fl/fl^ control BM-pDCs. Data are pooled from 5 independent experiments. **b**, Western blot of c-Jun protein in *c-Jun*^fl/fl^ and *c-Jun*^Δ/Δ^*Mx1*-Cre BM-pDCs stimulated with IMQ (16 h). **c**, *Gzmb* and *Trail* mRNA expression levels were analyzed by qRT–PCR in BM-pDCs stimulated with IMQ (4 h). (*Gzmb*: *c-Jun*^fl/fl^ *n* = 5 mice, *c-Jun*^Δ/Δ^*Mx1*-Cre *n* = 6 mice; *Trail*: LAL group *n* = 7 mice, IMQ group *n* = 8 mice; Data are pooled from 3 (*Gzmb*) and 4 (*Trail*) independent experiments). **d**, qRT–PCR detection of *Il6* mRNA (4 h) and ELISA detection of IL-6 protein (12 h) was performed on IMQ-stimulated BM-pDCs. (mRNA: *n* = 6 and IMQ: *c-Jun*^fl/fl^ *n* = 5, *c-Jun*^Δ/Δ^*Mx1*-Cre *n* = 7; Protein: *n* = 5 and *c-Jun*^Δ/Δ^*Mx1*-Cre: *n* = 7, IMQ *n* = 9; *n* is the number of mice pooled from 3 independent experiments). **e**, *Ifnb* mRNA and IFN- β protein levels were determined in BM-pDCs as described in (d). (mRNA: *n* = 7 and *c-Jun*^fl/fl^ *n* = 8, Protein: *n* = 5 and *c-Jun*^Δ/Δ^*Mx1*-Cre: *n* = 5, IMQ *n* = 9; *n* is the number of mice pooled from 4 independent experiments). **f**, pDC migration assay: BM-pDCs were allowed to migrate towards rCCL2 (3 h). **g**, Migration index of *c-Jun*^fl/fl^ and *c-Jun*^Δ/Δ^*Mx1*-Cre BM-pDCs in a migration assay performed as described in (f). (*n* = 4 per group and *c-Jun*^fl/fl^: *n* = 8, rCCL2 *n* = 4; *n* is the number of mice pooled from 5 independent experiments). **h**, Flow cytometric analysis of CCR2 expression on BM-pDCs. Left: Mean Fluorescence Intensity of CCR2. Right: Representative histograms. (*n* = 4 per group and *c-Jun*^fl/fl^: Isotype *n* = 10, CCR2 *n* = 9; *n* is the number of mice from 4 independent experiments). **i, j, k, l** Splenic pDCs (i) or lymph node pDCs (k) in *c-Jun*^fl/fl^ and *c-Jun*^Δ/Δ^*CD11c*-Cre mice treated with IMQ topically and orally (12 h) are shown in flow cytometry plots. Frequency of splenic pDCs (j) and lymph node pDCs (l) is shown among live, single cells. (*n* = 6 mice per group and *n* = 5 in the *c-Jun*^fl/fl^: IMQ group (j, l); *n* is the number of mice pooled from 2 (j, l) independent experiments). Data are shown as mean ± SEM. Dots in c-e, g, h, j and l represent biological replicates. *P*‐values were calculated by unpaired, two-tailed *t*-test (a), and two-way ANOVA with Tukey´s post-test (c-e, g, h, j and l). Source data

We proceeded to investigate the impact of c-Jun deletion on the functional properties of pDCs which are the primary source of IFN-I in our therapeutic approach. c-Jun is strongly induced in pDCs generated from FLT3L-supplemented bone marrow (BM-pDCs) upon IMQ treatment (Extended Data Fig. 8a,b). c-Jun-deficient BM-pDCs had a diminished expression of the pro-inflammatory cytokine IL-6, the type I IFNβ and the cytotoxic molecule granzyme B (Extended Data Fig. 8c–e). In contrast, other properties of pDCs remained unchanged. For instance, the migration of pDCs toward the chemokine CCL2 (Extended Data Fig. 8f–h) and the development of pDCs in the spleen and skin-draining lymph nodes (Extended Data Fig. 8i–l) were not affected by the absence of c-Jun.

Together, these results suggest that c-Jun in DCs is essential for the antitumor activity of IMQ, by at least two mechanisms: by modulating the recruitment of immune cells, such as pDCs, to the TME via CCL2 expression and by IL-12 expression.

### IL-12 inhibits tumor growth at the topically treated site

IL-12 can activate adaptive and innate immune cells^23^, and can inhibit tumor-associated angiogenesis^24,25^. As shown in Fig. 1e–h, the most striking histological features, in treated tumors, were the presence of vast necrotic areas and reduced numbers of blood vessels. The exact mechanism by which IL-12 inhibits angiogenesis is not well understood, but previous studies excluded a direct effect on endothelial cells^24,26^. Therefore, we investigated whether IL-12 can act directly on tumor cells and block angiogenesis. In vitro, IL-12 treatment significantly decreased VEGF-A production by B16-F10 cells (Fig. 6h) without affecting cell proliferation (Fig. 6i). IMQ itself did not impact VEGF-A production by tumor cells (Fig. 6h). To verify the functional relevance of IL-12 in tumor growth in vivo, we utilized an anti-IL-12 antibody to investigate its potential in counteracting the antitumor effect of IMQ. When IL-12 was neutralized the antitumor effect of topical and oral IMQ treatment was abolished and tumors had significantly smaller necrotic areas (Fig. 6j,k). These results suggest that IFNα-sensitized DCs produce more IL-12 in response to topical TLR7/8 agonists, in a c-Jun-dependent manner. IL-12 itself seems to be crucial for the antitumor effect and acts directly on tumor cells by reducing their VEGF-A production, leading to decreased vessel numbers and tumor necrosis.

### Combination therapy boosts antitumor immunity in metastases

We next explored the mechanisms leading to antitumor immunity at distant, nontreated metastases. Our previous findings had demonstrated that the rapid antitumor effect of IMQ in primary tumors is independent of T cells, NK cells and B cells^7^; however, stimulation of TLR7 can enable cross-presentation in DCs to expand the CD8^+^ T cell repertoire through a pathway that relies on type I IFN and the cytokine IL-12 (ref. ^27^).

**Fig. 7:**
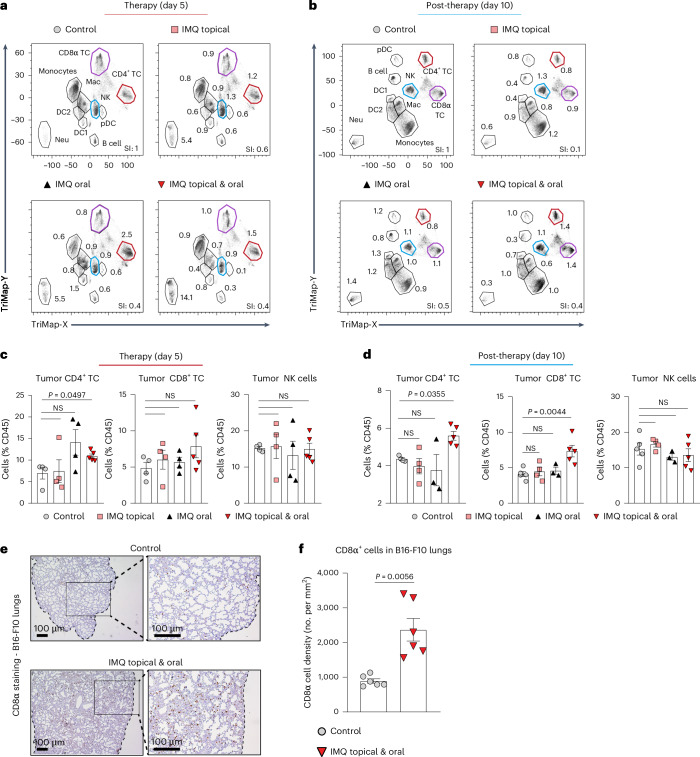
Combination therapy promotes a distant CD8^+^ T cell response. **a**, TriMap plot of immune cells (CD45^+^) in B16-F10 tumor-bearing mice (day 5) treated as indicated. Clusters are annotated in the top-left plot. The markers used were B220, BST-2, CD3, CD4, CD8a, CD11b, CD11c, CD64, CD103, Gr-1, Ly6-C, Ly6-G, MHC-II, NK1.1, TCRβ and XCR1. Differences in the immune cell composition to the control are shown as fold change for each cluster, and are summarized in the SI. *n* *=* 3 mice were concatenated per treatment condition. **b**, TriMap Plot of immune cells (CD45^+^) in B16-F10 tumor-bearing mice post-therapy (day 10) plotted as described in **a**. **c**, Frequency of CD4^+^ or CD8α T cells and NK cells was assessed by flow cytometry in B16-F10 tumors of mice (day 5) after treatment with IMQ: topical, oral and topical plus oral. *n* *=* 4 mice per group and *n* *=* 5 mice in the IMQ topical and oral group; data are from one experiment. **d**, Frequency of CD4^+^ or CD8α T cells and NK cells was analyzed by flow cytometry in B16-F10 tumors of mice (day 10) post-treatment with IMQ: topical, oral and topical plus oral. Control: *n* *=* 5, IMQ: topical *n* *=* 4, oral *n* *=* 3, topical and oral *n* *=* 5; *n* is the number of mice from one experiment. **e**, CD8α staining of B16-F10 colonized lungs at therapy end point (day 5) are shown. Metastasis was induced as described in Fig. 1i. Inset provides an enlarged view of the marked area. The black-dotted line shows the lung tissue border. Magnification, ×4 (left), ×10 (right). Scale bar, 100 µm. **f**, Enumeration of CD8α-positive cells in B16-F10 colonized lungs was performed by counting CD8α-stained sections, as shown in **e**. *n* *=* 6 mice per group; data are pooled from two independent experiment. Data are shown as mean ± s.e.m. Dots in **c**, **d** and **f** represent biological replicates. *P* values were calculated using unpaired, two-tailed *t*-test with Welch’s correction (**f**), Brown–Forsythe and Welch ANOVA test (**c**) and one-way ANOVA with Dunnett’s post-test (**d**). Source data

**Extended Data Fig. 9:**
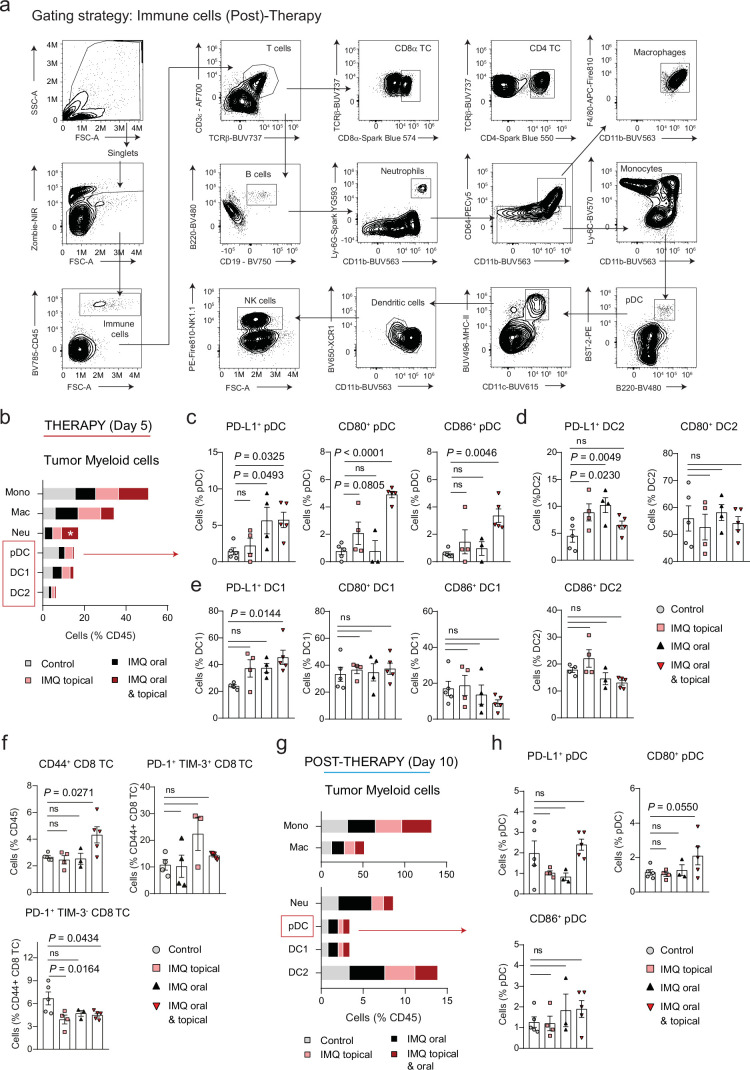
Myeloid cell composition in B16-F10 tumors after IMQ therapy. **a**, Flow cytometric gating strategy for lymphoid cells (see Fig. 7c, d) and myeloid cells (see Extended Data Fig. 9b–h). Cells were gated as follows: Within the viable cell gate: Immune cells (CD45^+^), T cells (CD3^+^, TCRβ^+^) CD4 or CD8α positive, B cells (-T cells, CD19^+^, B220^+^), Neutrophils (-B cells, Ly6-G^+^, CD11b^+^), Macrophages (-Neutrophils, CD64^+^, CD11b^+^, F4/80^+^), Monocytes (-Macrophages, Ly6-C^+^, CD11b^+^), pDCs (-Monocytes, BST-2^+^, B220^+^), DCs (-pDC, CD11c^+^, MHC-II^+^) XCR1 (Type I) or CD11b (Type II) positive and NK cells (-DC, NK1.1^+^). **b**, Stacked bar graph (horizontal) shows the frequency of myeloid cells in B16-F10 tumors after indicated treatment (Day 5). *P < 0.05. In b-e Control *n* = 5, IMQ: oral *n* = 4, topical *n* = 4, topical &oral *n* = 5; *n* is the number of mice from 1 experiment. **c**, Percentage of pDCs expressing PD-L1, CD80 or CD86, as assessed by flow cytometry in B16-F10 tumors treated as described in (b). **d**, Percentage of Type II DCs expressing PD-L1, CD80 or CD86, as assessed by flow cytometry at therapy end point (Day 5) as described in (b). **e**, Percentage of Type I DCs expressing PD-L1, CD80 or CD86, as assessed by flow cytometry, in B16-F10 tumors treated as described in (b). **f**, Frequency of CD8α T cells, positive for CD44 or PD-1 and TIM-3, was assessed by flow cytometry in B16-F10 at the ethical end point (Day 10). Mice were treated with IMQ orally or topically or both for 5 days. In f-h Control *n* = 5, IMQ: oral *n* = 3, topical *n* = 4, topical & oral *n* = 5; *n* is the number of mice from 1 experiment. **g**, Stacked bar graph (horizontal) shows the frequency of myeloid cells in B16-F10 tumors at the ethical end point (Day 10), as described in (f). **h**, Percentage of pDCs expressing PD-L1, CD80 or CD86, as assessed by flow cytometry in B16-F10 tumors treated as described in (f). Data are plotted as mean ± SEM. Dots in c-f and h represent biological replicates. *P*‐values were calculated using one-way ANOVA with Dunnett´s post-test (b-h). Source data

We, therefore, investigated the short-term (day 5; last day of therapy) and the long-term changes (day 10; 5 days post-therapy) in the tumor-immune infiltrate induced by our therapeutic regimen using flow cytometry (Extended Data Fig. 9a). At the therapy end point (day 5), we found pDCs to still be in an activated state (CD80, CD86 and PD-L1) (Extended Data Fig. 9b,c). Moreover, there was an increase in CD4^+^ T cells and neutrophils, but myeloid cells, including DCs and cytotoxic immune cells such as CD8^+^ T and NK cells, were unchanged (Fig. 7a–c and Extended Data Fig. 9d,e).

However, when we subsequently assessed lymphoid cells post-therapy (day 10), we found, in addition to CD4^+^ T cells, a significant increase in CD8^+^ T cells (Fig. 7b–d) with increased expression of the T cell-activation marker CD44 and decreased levels of the inhibitory checkpoint molecule PD-1 (Extended Data Fig. 9f). The myeloid cell compartment was not affected, and pDCs no longer exhibited an active cell state 5 days after treatment termination (Extended Data Fig. 9g,h).

We speculated that activation of the CD8^+^ T cell compartment could be responsible for the therapeutic effects of IMQ at nontreated metastatic lesions, as shown in Fig. 1i,j. Indeed, when we stained B16-F10 colonized lungs for CD8^+^ T cells, we found significantly higher numbers in treated mice compared to control mice (Fig. 7e,f), suggesting that IMQ combination therapy results in the generation of tumor-specific CD8^+^ T cells. These results indicate that our therapeutic approach promotes a CD8^+^ T cell response at distant sites.

**Fig. 8:**
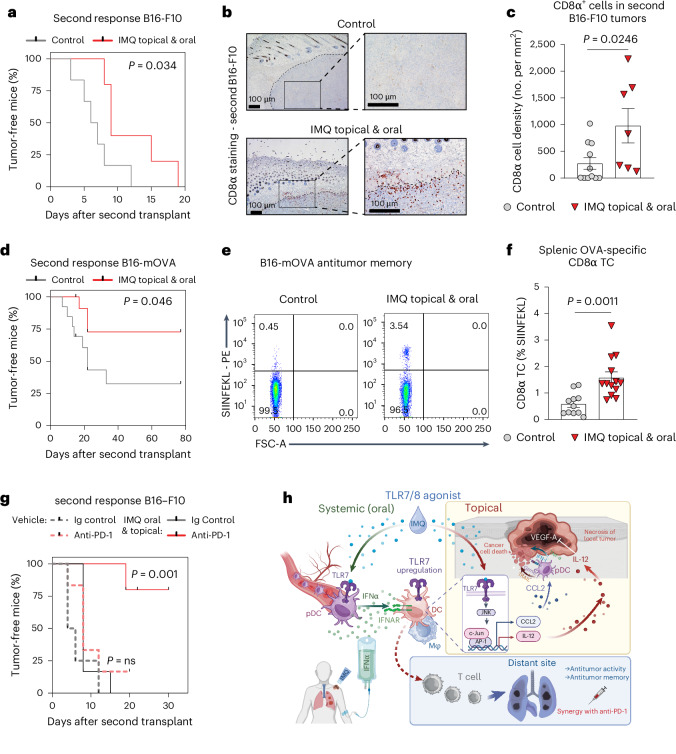
Combination therapy induces a memory CD8^+^ T cell response. **a**, Survival curve depicting the tumor-free time after tumor rechallenge with B16-F10 melanoma, following the memory model described in Extended Data Fig. 10a. Control *n* *=* 5, IMQ topical and oral *n* *=* 6; *n* is the number of mice pooled from two independent experiments. **b**, Immunohistochemistry CD8α staining in re-challenged B16-F10 tumors as described in **a**. The black-dotted line demarcates the tumor from the dermis. The inset gives an enlarged view. Magnification, ×4 (left), ×10 (right). Scale bar, 100 µm. **c**, Quantification of CD8α-positive cells from **b**. Control *n* = 11, IMQ topical and oral *n* = 7; *n* is the number of mice pooled from two independent experiments. **d**, Survival curve depicting the tumor-free time after tumor rechallenge with B16-mOVA melanoma. Control *n* = 13, IMQ topical and oral *n* = 13; *n* is the number of mice pooled from two independent experiments. **e**, Representative flow cytometry plots show SIINFEKL-positive CD8α T cells in the spleen 2 days after rechallenge with B16-mOVA. Cells were pre-gated on CD19^−^, TCRβ^+^, CD8α^+^. **f**, Quantification of splenic OVA-specific CD8α T cells from **e**. Control *n* = 11, IMQ topical and oral *n* = 13; *n* is the number of mice pooled from two independent experiments. **g**, Survival curve depicting the tumor-free time after tumor rechallenge with B16-F10 melanoma. Before resection mice received anti-PD-1 (200 µg) every other day (three times) and/or combination therapy (5 days). Ig control *n* = 4, anti-PD-1 *n* = 6; IMQ topical and oral: Ig control *n* = 6, anti-PD-1 *n* = 5; *n* is the number of mice pooled from two independent experiments. **h**, Graphical abstract: systemic IFN-I upregulates TLR7 expression on DCs/macrophages in the TME. Topical IMQ stimulates these sensitized cells to produce IL-12, which acts directly on tumor cells and blocks angiogenesis, resulting in necrosis. This two-hit treatment promotes CD8^+^ T cell antitumor immunity at distant sites, including memory formation and synergizes with PD-1 checkpoint blockade. Bar graphs are shown as mean ± s.e.m. Dots in **c** and **f** represent biological replicates. Survival curves are shown as percent, censored points only and no error bars. *P* values were calculated using unpaired, two-tailed *t*-test (**f**) and two-tailed Mann–Whitney *U*-test (**c**) or log-rank (Mantel–Cox) test (**a**,**d**,**g**). Source data

To further assess the potential of our combination therapy to induce a long-lasting tumor-specific CD8^+^ T cells response and promote memory development, we used B16-F10 and B16-mOVA (B16 cells expressing membrane-bound ovalbumin) melanoma cells in a model of tumor rechallenge that mimics tumor relapse in patients. For this purpose, mice with subcutaneous melanoma were treated with combined oral and topical IMQ (Extended Data Fig. 1a) followed by tumor resection. After 3 weeks, these mice were re-injected with the same tumor cell line. Interestingly, mice that were treated with oral and topical IMQ developed tumors significantly later than nontreated control mice (Fig. 8a). Moreover, significantly higher numbers of infiltrating CD8^+^ T cells were found in secondary tumors of mice whose primary tumors had been treated (Fig. 8b,c). The observed delay in tumor recurrence was limited in the B16-F10 melanoma model. We, therefore, decided to also use the B16-mOVA model, which is known to be more immunogenic and we observed protection from tumor rechallenge in 77% of IMQ-treated mice (Fig. 8d). Notably, these treated mice had an increase in OVA-specific CD8^+^ T cells in the spleen (Fig. 8e,f), demonstrating that melanoma specific memory T cells are formed with combination treatment.

**Extended Data Fig. 10:**
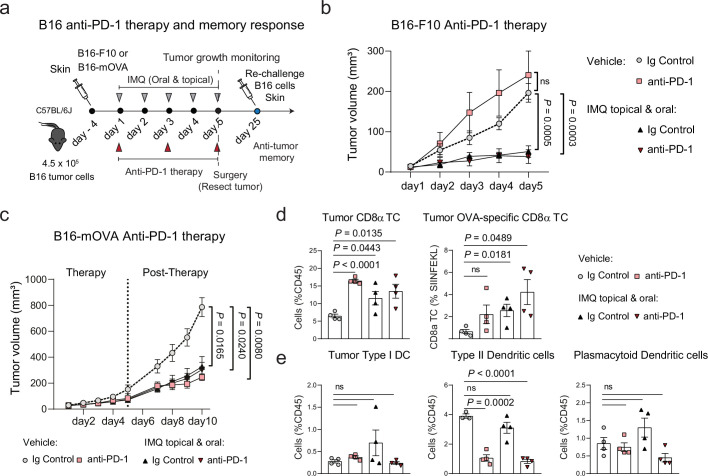
Antitumor effects of anti-PD-1 combined with IMQ on B16-F10 melanoma. **a**, Schematic representation of the murine model used to test the antitumor effect of anti-PD-1 therapy and memory formation after combination therapy. To test anti-PD-1 therapy mice were treated with anti-PD-1 antibody (200 µg, i.p.) every other day (3 times). To test memory formation: B16 tumors were surgically resected after five days of consecutive IMQ treatment (topical and oral), and mice were re-challenged at the indicated time-point. **b**, Tumor growth was monitored in B16-F10 melanoma-bearing mice. Mice were treated with anti-PD-1 antibody (every other day, 3 times), and/or IMQ topically and orally (5 consecutive days). (Vehicle: Ig Control and anti-PD-1 *n* = 6, IMQ topical &oral: Ig Control and anti-PD-1 *n* = 5; *n* is the number of mice from 1 experiment). **c**, Tumor growth was monitored in B16-mOVA melanoma-bearing mice during Therapy (5 days) and after treatment termination (post-Therapy). Therapy included treatment with anti-PD-1 antibody (every other day, 3 times), and/or IMQ topically and orally (5 consecutive days). In c-e *n* = 4 mice per group; 1 experiment. **d**, Frequency of CD8α T cells and of OVA-specific (SIINFEKL) CD8α T was assessed by flow cytometry in tumors described in (c). **e**, Percentage of Type I and Type II DCs and pDCs was analyzed by flow cytometry in tumors described in (c). Data are shown as mean ± SEM. Dots in d and e represent biological replicates. *P*‐values were calculated by unpaired, two-tailed *t*-test (d, e) and two-way ANOVA with Tukey´s post-test (b, c). Source data

Immune-checkpoint inhibitors have proven to be very effective in melanoma therapy^28^. Consequently, we investigated the potential synergistic effects of anti-PD-1 therapy, when combined with topical and oral IMQ treatment; however, in primary B16-F10 or B16-mOVA tumors, addition of anti-PD-1 to IMQ combination therapy did not further enhance local antitumor immunity (Extended Data Fig. 10a–c). Of note, anti-PD-1 therapy alone showed antitumor activity in the B16-mOVA, but not in the B16-F10 tumor model. An analysis of the immune cells in B16-mOVA tumors post-therapy (day 10) showed that our therapeutic approach was as effective as anti-PD-1 therapy (alone or in combination with IMQ) at inducing CD8^+^ T cells and generating OVA-specific CD8^+^ T cells in the primary tumor (Extended Data Fig. 10d). Notably, plasmacytoid DCs were unaffected by anti-PD-1 therapy, but type II DCs were markedly reduced (Extended Data Fig. 10e).

We next explored the synergism between anti-PD-1 therapy and IMQ combination therapy in the B16-F10 antitumor memory model. We found that in the poorly immunogenic B16-F10 tumor model, where TRL7/8 agonist treatment could delay, but not prevent the appearance of tumors after rechallenge (Fig. 8a), the addition of anti-PD-1 antibody resulted in complete protection from tumor relapse in 80% of the mice (Fig. 8g). Conversely, PD-1-blockade alone had no effect on the secondary response to B16-F10 tumors. These results demonstrate that a combination therapy utilizing topical and oral IMQ effectively induces a systemic CD8^+^ T cell-dependent immune response, specifically targeting tumor antigens. This immune response confers protection against lung metastases and tumor relapses and therapeutic efficacy can be further augmented through synergistic treatment with anti-PD-1 antibodies.

## Discussion

Topical IMQ is currently approved for the treatment of actinic keratosis^4^ and basal cell carcinoma^5^, but its efficacy in melanoma patients is poor. IMQ is administrated directly to the lesions and, in contrast to mice, its systemic absorption is minimal in patients^29^. Here, we show in preclinical tumor models that simultaneous topical and oral IMQ administration has a much stronger antitumor effect and that this treatment strategy effectively treats melanoma and breast cancer, potentially also in patients who do not respond to immunotherapy. The antitumor effects we find are not confined to the locally treated tumors, but are also seen in distant metastases. Furthermore, the treatment leads to the development of an antitumor memory, which in turn protects from further metastases and relapses (Fig. 8h).

Mechanistically, oral IMQ activates pDC to produce IFN-I, which then exerts a profound impact on the tumor microenvironment (TME) by inducing TLR7/8 expression on DCs and macrophages in both mice and in patients. These primed DCs produce IL-12 in response to topical IMQ treatment through TLR7–c-Jun signaling, which then blocks angiogenesis and induces tumor necrosis at the local sites (Fig. 8h). Of note, the effects of IMQ, seen in mice, depend only on TLR7 as TLR8 is nonfunctional and thus nonresponsive to IMQ^30,31^. IFNα can induce TLR7 expression on DC1 (ref. ^32^) and TLR7-expressing cells, such as DCs, produce IL-12 upon stimulation with TLR7/8 agonists^33^. Our results are in line with these findings and further explore the therapeutic applications of these effects.

We already reported that the development and function of DCs depends on the AP-1 transcription factor c-Jun^34^. Here, we identify c-Jun as a central player in DCs mediating the antitumor effect of IMQ through two mechanisms: by controlling tumor angiogenesis via IL-12 expression and pDC recruitment via CCL2. We discover that the expression of both IL-12 composing subunits, IL-12A and IL-12B, is dependent on c-Jun/AP-1 and that pDC infiltration into the TME of mice lacking c-Jun in DCs is strongly impaired. Similar pDC recruitment defects are seen in mice lacking the chemokine CCL2 and the role of c-Jun in CCL2 regulation has been shown in fibroblasts^35^ and DCs^10^.

While IL-12 has already demonstrated robust antitumor activity in preclinical models, its clinical translation has been hindered by challenges associated with its instability and short half-life^23^. Current approaches, such as IL-12 plasmid electroporation^36^ or adoptive transfer of IL-12-producing cells, aim to deliver IL-12 directly to the tumor site to overcome these limitations^37^. Although further experiments in genetic mouse models are needed to unequivocally show that IL-12 derived from DCs is critical for the antitumor response, we demonstrate that our treatment approach with topical IMQ offers an alternative strategy for endogenous IL-12 production within the TME. IL-12 could also be involved in the formation of tumor-specific CD8^+^ T cells through the induction of tumor necrosis. This tumor cell death likely provides an immunogenic environment that promotes the activation of DCs through danger-associated molecular patterns^38^ and the release of neo-antigens to exert an in situ cancer vaccine-like effect^39^.

Our model is in line with previous studies that show that TLR7 stimulation activates cross-presentation in DCs^27^ and that pDCs have potent T cell stimulatory capacities through IFN-I production^40^. IFN-I has known antitumor properties and can promote the maturation and activation of DCs, required for CD8^+^ T cell-dependent tumor rejection. Previous studies also showed that CD4^+^ T cells can promote an effector and memory CD8^+^ T cell response^41,42^, and that CD4^+^ T cells can exhibit direct antitumor immunity^43^. We observed a c-Jun/CCL2-dependent infiltration of CD4^+^ cells in treated tumors, which could potentially contribute to the observed antitumor immunity. Myeloid cell frequency, except for neutrophils, was unchanged in treated tumors. Neutrophils are described to have a dual role in tumor immunity^44^ and have the potential to activate T cells^45^. Further studies are needed to address the role of different myeloid cells in IMQ antitumor effect.

The memory response evoked by topical and oral IMQ is potentiated by concomitant PD-1 checkpoint immune therapy in both highly and poorly immunogenic tumor models. This is in line with current clinical trials investigating the efficacy of anti-PD-1 antibodies when combined with immune stimulators of the TLR family^46,47^. Of note, the combination of anti-PD-1 with IMQ did not improve the antitumor effect in primary tumors, which was expected considering our results demonstrating a predominant effect of innate immunity in local sites. In fact, we have previously shown that the IMQ antitumor effect at the primary tumor site is independent of adaptive immune cells^7^. We reveal a dual and additive function of pDCs in the antitumor immunity induced by IMQ. First, when IMQ is administered orally, pDCs contribute to the generation of systemic IFN-I. Second, when IMQ is administered topically, it activates pDCs to unleash cytotoxic molecules that exert localized tumor cell killing within the TME, as we have previously shown^7^.

Furthermore, we confirm that our therapeutic regimen is effective not only in melanoma but also in preclinical breast cancer models, including the MMTV-PyMT transgenic model. Indeed, previous studies have shown that treatment strategies invoked by our therapeutic approach such as blocking angiogenesis^48^, delivery of IL-12 (ref. ^49^) or stimulation of CD8^+^ T cells^50^ effectively reduces mammary cancers in this mouse model of breast cancer.

Of note, our results suggest that oral IMQ can be substituted by systemic IFN-I, which is already widely used in clinics. In patients with cancer, the use of systemic IFN-I has been until now limited by its adverse events, which include bone-marrow aplasia and depression^51^. These toxicities correlate with dosage and length of treatment^52,53^. Our proposed regimen would involve a brief treatment with IFN-I, thereby making such adverse events less likely. To overcome the possible deleterious effects of systemic IFN-I, other groups investigated the use of IFN-I or stimulators of IFN genes (e.g. STING agonists) in formulations that ensure targeted delivery to DCs, which limits systemic inflammation and the associated adverse events^54,55^. In our hands, a short treatment with systemic IFN-I and topical TLR7/8 agonist was capable of inducing a long-lasting immune response in primary tumors and at distant sites, which are not treated with IMQ, without evident toxicity in mice.

In conclusion, we present a therapeutic regimen based on topical IMQ and systemic IFN-I for the treatment of melanoma, and potentially other topically accessible cancers, such as breast cancer. Our combined treatment exploits the immunostimulatory properties of both IFNα and TLR7/8 signaling and is effective in the treatment of primary tumors, while protecting form metastasis and tumor relapse by inducing immunological memory (Fig. 8h). These findings hold great promise for improving the outcomes of patients with topical accessible cancers in the near future.

## Methods

### Ethical regulations

We confirm that the research performed in the present study complies with all ethical regulations. All animal experiments conducted in this study were performed in accordance with the federal laws and guidelines of the Medical University of Vienna. The animal experimental procedures were approved by the Animal Experimental Ethics Committee of the Medical University of Vienna and the Austrian Federal Ministry of Science and Research (animal license nos. BMBWF-66.009/0200-WF/II/3b/2014 and BMBWF-66.009/0319-V/3b/2019). The retrospective analysis of IFN-I-treated patients with melanoma from Klinik Landstraße, Austria conformed to the principles set out in the WMA Declaration of Helsinki. All samples were obtained through informed consent and are in accordance to the principles of good clinical practice and the study guidelines provided by the Department of Dermatology at the Klinik Landstraße, Austria.

### Animals

Mice were housed in the animal facility of the Medical University of Vienna and had unlimited access to standard laboratory chow and water under a light–dark cycle of 12 h and a housing temperature of 22 ± 1 °C. Up to three mice were kept in a cage. Female or male mice at 8–12 weeks old, were used. The maximum tumor burden of 2,000 mm³ was not exceeded and tumor-bearing mice with ulcerated tumors were immediately killed. C57BL/6 and BALB/C mice were purchased from Harlan Laboratories and Janvier Labs, respectively. *Tlr7*^−/−^ (ref. ^3^), *Ifnar*^−/−^ (ref. ^17^), *Bdca2*-DTR^56^, *c-Jun*^fl/fl^ (ref. ^57^) mice crossed to *CD11c*-Cre, or *Mx1*-Cre^58^ mice were bred in house.

### Cell lines

The B16-F10 cell line was purchased from the American Type Culture Collection (CRL-6475; RRID: CVCL_0159) and cultured in RPMI 1640 medium in the presence of 10% FCS (PAA), 1% penicillin and streptomycin (pen–strep) (PAA), 1% sodium pyruvate, 2 mM glutamine, 1% non-essential amino acids and 50 mM β-mercaptoethanol. B16-mOVA cells were a generous gift of T.F. Tedder (Duke University), and they were cultured in DMEM containing 10% FCS (PAA), 1% pen–strep (PAA), 4 nM glutamine, 50 mM β-mercaptoethanol and 400 μg ml^−1^ G418 (Roche). The murine breast cancer cell line 4T1 was kindly provided by A. Csiszar (Medical University of Vienna).

### Tumor models

To induce orthotopic tumors in mice cancer cells were collected at 70–80% confluency by trypsinization (Sigma-Aldrich), washed twice and resuspended in PBS to the desired cell concentration, before injection. Orthotopic melanoma tumors were induced in the shaved back skin of female and male mice, 8–12 weeks of age, by intradermal injection of 4.5 × 10^5^ B16-F10 cells or B16-mOVA cells. Lung metastases were induced by injection of 4.5 × 10^5^ B16-F10 cells into the tail vein. Simultaneously, B16-F10 cells were injected into the skin. Two-flank melanoma tumors were induced by inoculation of the left and right flank with 4.5 × 10^5^ B16-F10 cells in the shaved back skin.

Orthotopic breast cancer tumors were induced in female BALB/C mice by injecting 1 × 10^6^ tumor cells into the right-side fat pad of the fifth pair of mammary glands.

Antitumor memory was induced as follows: primary melanoma tumors were induced and treated with IMQ as described, followed by surgical resection of the tumor at the therapy end point. After 20 days, tumor-free mice were re-injected with 4.5 × 10^5^ B16-F10 or B16-mOVA cells. Mice were killed after tumors re-grew. For the detection of OVA-specific CD8^+^ T cells in the B16-mOVA rechallenge tumor model, mice were killed 2 days after rechallenge and spleens were collected for analysis by flow cytometry.

To assess tumor growth, length and width were measured daily during the treatment period by an investigator who was not blinded. After treatment termination, tumor size was monitored until reaching the ethical end point. Tumor volume was calculated using the ellipsoidal formula: length × width^2^ × π/6.

### TLR7/8 agonist therapy

Therapy was started when the tumors (B16-F10 or 4T1) reached an average size of 50 mm^3^. The tumor-bearing mice were then randomly assigned to the different treatment groups and were housed individually, with one mouse per cage wearing a collar.

IMQ was administered topically on the primary tumor and/or orally using 20 mg of Aldara cream (5% imiquimod; MEDA), which corresponds to 1 mg IMQ per treatment (topical or oral), for 5 consecutive days. For the oral treatment the mice licked the cream from the tip of a 1-ml syringe. To prevent unintended ingestion of IMQ, Elizabethan collars from Harvard Apparatus were used.

For R848 therapy, mice received IMQ topically and Resiquimod (Invivogen) orally (100 µg, oral gavage) for 5 consecutive days.

### In vivo mouse treatment

IFN-I treatment was performed by intraperitoneal (i.p.) injection of 10,000 U of recombinant mouse Interferon Alpha A (12100-1; PDL Assay Science) per day.

Depletion of pDCs was induced by diphtheria toxin from *Corynebacterium* *diphtheria* (D0564, Sigma) injection (i.p.; 4.5 ng DT g^−1^ every day) in *Bdca2*-DTR mice, starting 1 day before IMQ treatment.

Blocking of IL-12 was achieved by delivering 500 µg neutralizing anti-IL-12B antibody daily (i.p., clone 17.8, BE0051, Bio X Cell). In the control group, mice were administered an Ig antibody daily (i.p., clone 2A3 BE0089, Bio X Cell). Treatment started on the first day of IMQ administration.

For the anti-PD-1 antibody experiments, mice were treated with 200 μg anti-PD-1 antibody (clone 29F.1A12, BP0273, Bio X Cell) or Ig control antibody (clone 2A3, BE0089, Bio X Cell) on day 1, 3 and 5 of the IMQ treatment.

### MMTV-PyMT breast cancer model

MMTV-PyMT mice were purchased from Jackson Laboratories (strain 002374, RRID:IMSR_JAX:002374). Female mice developed mammary cancers in the cervical glands at 8 weeks of age. As a therapy, mice received IMQ orally and topically for 5 consecutive days, followed by an additional 5 days of treatment after a 5-day break. Only the cervical mammary glands were treated topically with IMQ. All the mammary glands were palpated to evaluate the tumor burden. A palpable mass >3.5 mm² was considered a mammary cancer and tumor growth was measured with a caliper. At 11 weeks of age MMTV-PyMT mice reached the ethical end point defined as a cumulative tumor size <2,000 mm³.

### Bioluminescent imaging

In vivo bioluminescent imaging of metastatic lungs was carried out on a Lago X imaging system (Spectral Instruments Imaging). Acquisition parameters were 60 s exposure time; binning, 16; and FStop, 1.2. Images were analyzed using Aura software (v.4.0.8). Background-corrected region of interest measurements of the lung were conducted.

### Histology and immunohistochemistry

Paraffin embedding was performed for mouse samples, and both mouse and patient samples were sectioned into 5-μm slices for subsequent analyses. Standard procedures were followed for hematoxylin and eosin (H&E) (Sigma-Aldrich) and immunohistochemistry (IHC) staining.

IHC was conducted using the following antibodies: anti-mouse endomucin (Thermo Fisher Scientific, 12-5851-80, RRID:AB_891531), recombinant anti-CD8α (Abcam, ab217344, RRID:AB_2890649), anti-TLR7 (Abcam, ab124928, RRID:AB_11131208) and anti-TLR8 (Abcam, ab53630, RRID:AB_883061) (Supplementary Table 2).

Images were recorded on a Nikon Eclipse 80i microscope and analyzed with ImageJ (RRID:SCR_003070; http://imagej.nih.gov/ij) or Adobe Photoshop (RRID:SCR_014199; Adobe Systems).

Tumor necrosis was assessed in ×4 images, blood vessels were quantified in ×20 images (three per mouse), lung metastases was counted in ×10 images (five per mouse) and CD8^+^ T cell counts were obtained from ×20 images (three per mouse). Cell counts are presented as cell density (number of cells per mm²).

### Multiplex immunofluorescence

Paraffin sections with a thickness of 4 µm were prepared for multiplex immunofluorescence by deparaffinizing them through incubation at 65 °C for 15 min, followed by rehydration using an alcohol series according to standard protocols. Samples were blocked (2% BSA, 5% horse-serum and 0.1% Triton in PBS) to prevent nonspecific binding.

To detect multiple tissue markers, we used the Opal 6-Plex Detection kit (NEL811001KT, Akoya Biosciences) according to the manufacturer’s instructions. Briefly, we incubated the samples with primary antibodies at room temperature (RT) for 1 h, followed by an incubation with the secondary HRP-coupled antibody (10 min, RT). To generate the Opal signal the slides were then stained with the appropriate Opal Fluorophore (10 min, RT). Microwave treatment was used to remove the primary–secondary HRP complex. This process was repeated four more times to detect five tissue markers (Supplementary Table 3). Finally, the samples were counterstained with 4,6-diamidino-2-phenylindole (DAPI) to visualize the nucleus. Images were recorded on a Slide Imaging System (Vectra Polaris, Akoya), processed with Phenochart Whole Slide Viewer (Akoya, v.1.1.0) and Inform Tissue Analysis Software (Akoya, v.2.6) and analyzed using QuPath (v.0.4.3) and HALO (Indica Labs, v.3.5.3577.214).

### In vitro BM-DC culture

Bone marrow (BM) was collected from the femur and tibia. The BM was then passed through a 70-µm cell strainer and red blood cells were lysed using ACK lysis buffer (420301, BioLegend).

To generate DCs and pDCs, BM cells were seeded at a concentration of 4.5 × 10^6^ cells per ml and cultured for 7 days in the presence of 80 ng ml^−1^ FLT3L (250-31L, Prepotec) in a cell culture medium containing RPMI (22400-089, Gibco) supplemented with 10% FCS (F9665, Sigma), 1% glutamine (G7513-100ML, Sigma), 1% non-essential amino acids (11140-035, Gibco), 1% sodium pyruvate (S8636-100ML, Sigma), 0.1% β-mercaptoethanol (31350-010, Gibco) and 1% Pen–Strep (P4333-100ML, Sigma) as previously described^59^.

BM-DCs were separated from BM-pDCs by magnetic sorting using a biotinylated anti-B220 antibody (103204, BioLegend, RRID:AB_312989) and streptavidin magnetic beads (557812, BD Biosciences). The positively (BM-pDC) and/or the negatively enriched fraction (BM-DC) were utilized for further experiments. Both fractions exhibited a purity >80%.

### In vitro BM-macrophage culture

To generate BM-macrophages, BM cells were cultured for 7 days in complete medium: DMEM F-12 containing 10% FCS (F9665, Sigma), 2 mM l-glutamine (G7513-100ML, Sigma), 100 U ml^−1^ penicillin and 100 µg ml^−1^ streptomycin (P4333-100ML, Sigma) and 20% L929 conditioned medium. On day 3 of culture, 5 ml of fresh complete medium was added and the medium was changed completely on days 5 and 7.

### In vitro stimulation

For in vitro stimulation experiments, BM-DCs and BM-macrophages were plated at a density of 2.5 × 10^5^ cells per well, while BM-pDCs were seeded at a density of 1 × 10^6^ cells per well.

The seeded cells were stimulated with the following agents for varying time points: 500 U ml^−1^ of recombinant mouse Interferon Alpha A (12100-1, PDL Assay Science) and 2.5 µg ml^−1^ of Imiquimod (R837, tlrl-imq, Invivogen).

### Luminex and ELISA

Luminex measurements were conducted on plasma, cell culture supernatant and protein extracted from tissues (100 μg per 50 μl) using the ProcartaPlex Mouse IFN α/β panel (EXP020-22187-901, Thermo Fisher Scientific) as well as customized panels for mouse VEGF-A (Thermo Fisher Scientific). Data were acquired using a Bio-Plex MAGPIX system (Bio-Rad).

Culture supernatant from in vitro generated BM-DCs and macrophages was collected, and the levels of IL-12 and CCL2 (MCP-1) were measured using IL-12 (p70) (433604; BioLegend) and MCP-1 (432704; BioLegend) ELISA kits according to the manufacturer´s instructions.

### Western blot

Western blot was done as previously described^7^. The following antibodies were used: mouse c-Jun rabbit antibody (clone 60A8, 9165, Cell Signaling, 1:500 dilution) and mouse Vinculin mouse antibody (clone hVIN-1, V9131, Sigma-Aldrich, 1:500 dilution).

### Migration assay

Purified BM-pDCs were seeded at a density of 1 × 10^6^ cells in the upper chamber of a Transwell insert (6.5-mm diameter) with 5-μm membrane pores (Sigma- Aldrich). Migration was induced by recombinant murine CCL2 (500 ng, 578406, BioLegend) in the lower chamber for 3 h at 37 °C. Migrated cells were analyzed by flow cytometry using 123count eBeads (01-1234-42, Thermo Fisher) for cell counting. The migration index was calculated as number of cells in sample/number of cells in control.

### Cell purification and flow cytometry

Tumors were isolated, cut into pieces and enzymatically digested using 100 µg ml^−1^ Liberase (Roche) and 100 µg ml^−1^ DNase I (Sigma) for 45 min at 37 °C. After digestion, the cells were washed and filtered through a 70-µm cell strainer to obtain a single-cell suspension.

Lymph nodes or spleen were sheard with scissors, incubated for 15 or 30 min in a digestion buffer (PBS with Ca^2+^ and Mg^2+^) that contained Liberase (100 µg ml^−1^) and DNase I (100 µg ml^−1^) at 37 °C. To generate a single-cell suspension cells were filtered through a 70-µm cell strainer. Spleen red blood cells were lysed with RBC lysis buffer (BioLegend).

Unspecific binding of antibodies was blocked by incubation of cells with anti-mouse CD16/32 antibody for 10 min at 4 °C (clone S17011E, 156604, BioLegend). Subsequently cells were stained with fluorescently labeled antibodies (Supplementary Table 4) for 30 min at 4 °C. Stained cells were washed, filtered and recorded on a LSR Fortessa cell analyzer (BD Biosciences, RRID:SCR_018655) or a Cytek Aurora Spectral Analyzer (Cytek Biosciences, RRID:SCR_019826).

### SIIN-tetramer staining

For the flow cytometric detection of OVA-specific T cells, R-PE labeled Pro5 MHC pentamer antibody specific for H-2Kb SIINFLEKL was used according to manufacturer’s instructions (F093-2A-E, Proimmune). In brief, Pro5 MHC pentamer antibody was added for 10 min at RT to cells isolated from tumor or spleen (2 × 10^6^). After incubation, cells were washed with FACS buffer and stained before analysis.

### Intracellular cytokine staining

Cells were fixed using the Cyto-Fast Fix/Perm Buffer Set (426803, BioLegend) when staining for cytokines or intracellular receptors, following the instructions of the manufacturer. For cytokine detection, Brefeldin A (420601, BioLegend) was added to all buffers before fixation. Zombie Aqua Fixable Viability kit (423101, BioLegend) was used to stain dead cells. Fixed cells were washed twice with FACS buffer and Cyto-Fast Perm Wash Solution (BioLegend), stained for intracellular proteins (Supplementary Table 4) for 45 min at RT and then washed twice with Cyto-Fast Perm Wash Solution and FACS buffer before flow cytometric analysis.

### Cell sorting

Sorting of stained cells was performed on a FACS Aria III Cell sorter (BD Biosciences, RRID:SCR_016695). Cells were sorted into TRIzol LS Reagent (10296028, Thermo Fisher Scientific). For sorting of myeloid cells, cells were pre-gated on live, singlets, CD45^+^, CD3^−^, CD19^−^, NK1.1^−^. Tumor-infiltrating DCs (CD45^+^CD64^−^CD11c^+^MHC-II^+^) and tumor-infiltrating macrophages (CD45^+^CD64^+^CD11b^+^) were sorted.

### Flow cytometry data processing and visualization

Collected flow cytometric data was analyzed using FlowJo software (v.10.9.0, RRID:SCR_008520). For the multidimensional *t*-distributed stochastic neighbor embedding and Uniform Manifold Approximation and Projection (UMAP) analysis, we utilized the following plugins obtained from FlowJo Exchange (https://www.flowjo.com/exchange/#/): DownSample v.3.3.1, UMAP v.4.0.3 and TriMap v.0.2. Before dimensionality reduction of the data, samples were pre-gated on live, single CD45^+^ cells and cell numbers were adjusted to be equal. Samples belonging to the same group/condition were concatenated. Immune clusters identified by dimensionality reduction were confirmed by classical gating.

The Similarity Index (SI) identifies, for every cluster annotated, the group/condition with the maximum distance (0) to the control. Cluster values in the groups are between 1 (control) and 0, and the mean calculated equals the SI.

For the visualization of flow cytometry data in heatmaps and dot plots, we used the R packages pheatmap (v.1.0.12) and ggplot2 (v.3.4.3), respectively. As an input, the fold change (group/condition versus control) was calculated.

### RNA isolation and gene expression analysis

RNA from cells was isolated using the standard TRIzol extraction protocol (Thermo Fisher) and complementary DNA synthesis was performed using the SuperScript First-Strand Synthesis System (Invitrogen) following the manufacturer’s instructions. Similarly, RNA from sorted cells was isolated using the miRNeasy kit (217084, QIAGEN) and cDNA synthesis was performed using the SuperScript IV Reverse Transcriptase enzyme (15307696, Invitrogen).

Quantitative PCR with reverse transcription (RT–qPCR) reactions were performed using the SYBR Green Mix reagent (Applied Biosystems) on a C1000 Touch Thermal Cycler equipped with a CFX96 Real-Time System (Bio-Rad). Primers used in this study are listed in the Supplementary Table 5.

Relative quantification of RNA was calculated by the ΔΔ Ct method. Expression levels were normalized to the reference gene TATA-binding protein (*Tbp*).

### scRNA-seq data and analysis

scRNA-seq analysis was conducted on a publicly available dataset^19^ (GSE150361) focusing specifically on wild-type mice that were either untreated or treated with IMQ. The dataset was processed using the Seurat pipeline^60^. Cells were excluded if the number of detected genes fell outside the range of 200 to 4,000 or if the percentage of mitochondrial genes exceeded 7.5%. Clustering was performed using the FindNeighbors and FindClusters functions with 20 dimensions and a resolution of 0.5, respectively.

### TCGA melanoma data and analysis

Clinical data and mRNA sequencing data of the 470 patients with melanoma included in the TCGA–SKCM project were obtained from the official websites of the project (http://cancergenome.nih.gov and https://portal.gdc.cancer.gov/). Among these patients, information on previous IFNα treatment was available for a subset of 43 patients, who were selected for further analysis.

### Patient samples

Patient samples have been obtained as part of routine diagnosis and were treated according to national guidelines in the Department of Dermatology of the Klinik Landstrasse. All procedures were in accordance with the ethical standards of the institution. All patients signed a written informed consent before biopsy. Paraffin-embedded samples of patients who received IFN-I therapy were retrospectively selected and used for multiplex immune-fluorescence. Patient samples were anonymized and only the treatment status was known. None of the patients received compensation.

### Statistics and reproducibility

Statistical analysis was conducted using GraphPad Prism (v.8, RRID:SCR_002798). An unpaired, two-tailed *t*-test was employed to compare the means between two experimental groups. In cases where the s.d. were unequal, a Welch’s *t*-test was utilized.

For experiments involving more than two groups, statistical analysis was conducted using one-way analysis of variance (ANOVA) followed by a Tukey multiple comparison post hoc test. A two-way ANOVA was employed to compare the tumor growth curves and to assess statistical differences between groups with multiple parameters. A Spearman’s rank correlation test was used to analyze the correlations between variables. A log-rank (Mantel–Cox) test was employed to compare tumor-free mice after rechallenge (survival curves). Data distribution was assumed to be normal but this was not formally tested.

Data are presented as the mean ± s.e.m. of at least two independent pooled experiments with at least three biological replicates per group, unless otherwise specified. The exact *P* values are reported in each figure legend. Data were excluded if a mathematical outlier was identified using the ROUT (multiple) or Grubbs’ test (one) in GraphPad. Animals were excluded from experiments if they died or had to be killed to comply with ethical regulations.

No statistical methods were used to pre-determine sample sizes but they were chosen to be similar to those reported in previous publications for the same type of experiments^7^. Age- and sex-matched mice were randomly allocated to experimental groups. The investigators were not blinded during experiments and outcome assessment.

### Reporting summary

Further information on research design is available in the Nature Portfolio Reporting Summary linked to this article.

## Supplementary information

Supplementary Information Supplementary Figs. 1 and 2.

Reporting Summary

Supplementary Tables Supplementary Table 1. List of correlation of TLR7/8 with different cell type markers in a TCGA-SCKM patient subset that received IFN-α. Supplementary Table 2. List of murine markers to characterize the tumor microenvironment by IHC. Supplementary Table 3. List of human markers used to stain myeloid cells in patients with melanoma by multiplex immunofluorescence. Supplementary Table 4. List of antibodies used to determine immune cells by flow cytometry. Supplementary Table 5. Primer sequences for gene expression analysis by RT–qPCR.

## Source data

Source Data Fig. 1 Statistical source data.

Source Data Fig. 2 Statistical source data.

Source Data Fig. 3 Statistical source data.

Source Data Fig. 4 Statistical source data.

Source Data Fig. 5 Statistical source data.

Source Data Fig. 6 Statistical source data.

Source Data Fig. 7 Statistical source data.

Source Data Fig. 8 Statistical source data.

Source Data Extended Data Fig. 1 Statistical source data.

Source Data Extended Data Fig. 2 Statistical source data.

Source Data Extended Data Fig. 3 Statistical source data.

Source Data Extended Data Fig. 4 Statistical source data.

Source Data Extended Data Fig. 6 Statistical source data.

Source Data Extended Data Fig. 7 Statistical source data.

Source Data Extended Data Fig. 8 Statistical source data.

Source Data Extended Data Fig. 9 Statistical source data.

Source Data Extended Data Fig. 10 Statistical source data.

Imaging Source Data Unprocessed blots.

## Data Availability

This study includes no data deposited in external repositories. Previously published mRNA and clinical data of the TCGA–SKCM project have been reanalyzed in this study and are available on the official websites of the project (http://cancergenome.nih.gov and https://portal.gdc.cancer.gov/). Gene expression data of Tlr7 were retrieved from the Immune Dictionary Portal (https://www.immune-dictionary.org/app/home) and the Immunological Genome Project (https://rstats.immgen.org/Skyline_microarray/skyline.html?datagroup=IFN). Previously published scRNA-seq data of mouse IMQ-inflamed skin that were reanalyzed here are available in the Gene Expression Omnibus under accession code GSE150361 (ref. ^19^). Source data for Figs. 1–8 and Extended Data Figs. 1–10 are provided with the paper. All other data supporting the findings of this study are available from the corresponding author on reasonable request.

## Code availability

No custom algorithms were generated for this study. Data analysis was conducted using published software packages as detailed and referenced in the Methods.
