# Supplementary material for: Systemic IFN-I combined with topical TLR7/8 agonists promotes distant tumor suppression by c-Jun-dependent IL-12 expression in dendritic cells
Source: Nat Cancer. 2025 Jan 23;6(1):175–93. doi: 10.1038/s43018-024-00889-9 (PMC11779648; doi:10.1038/s43018-024-00889-9)
Supplement: Supplementary file 1 — Supplementary Figs. 1 and 2. [file 43018_2024_889_MOESM1_ESM.pdf]

nature cancer

Supplementary information https://doi.org/10.1038/s43018-024-00889-9

Systemic IFN-I combined with topical TLR7/8 agonists promotes distant tumor suppression by c-Jun-dependent IL-12 expression in dendritic cells

In the format provided by the authors and unedited

Supplementary Figure 1 Multiplex IF staining for myeloid cells in melanoma patients.

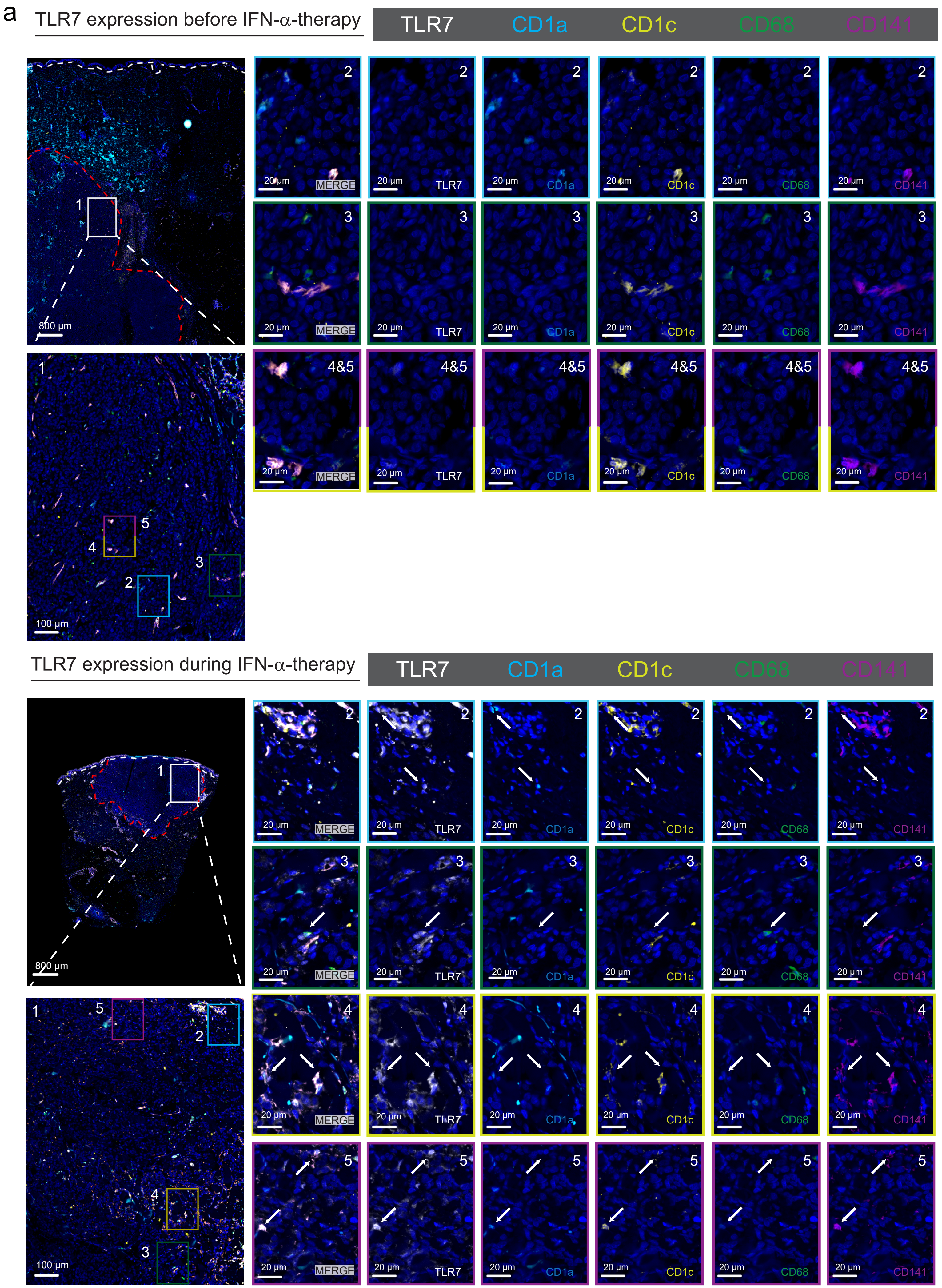

a, Single-color images for each inset are shown for the mulitplex staining described in Fig.4a.

Supplementary Figure 2 Multiplex-IF staining to characterize TLR7 expression on myeloid cells.

a

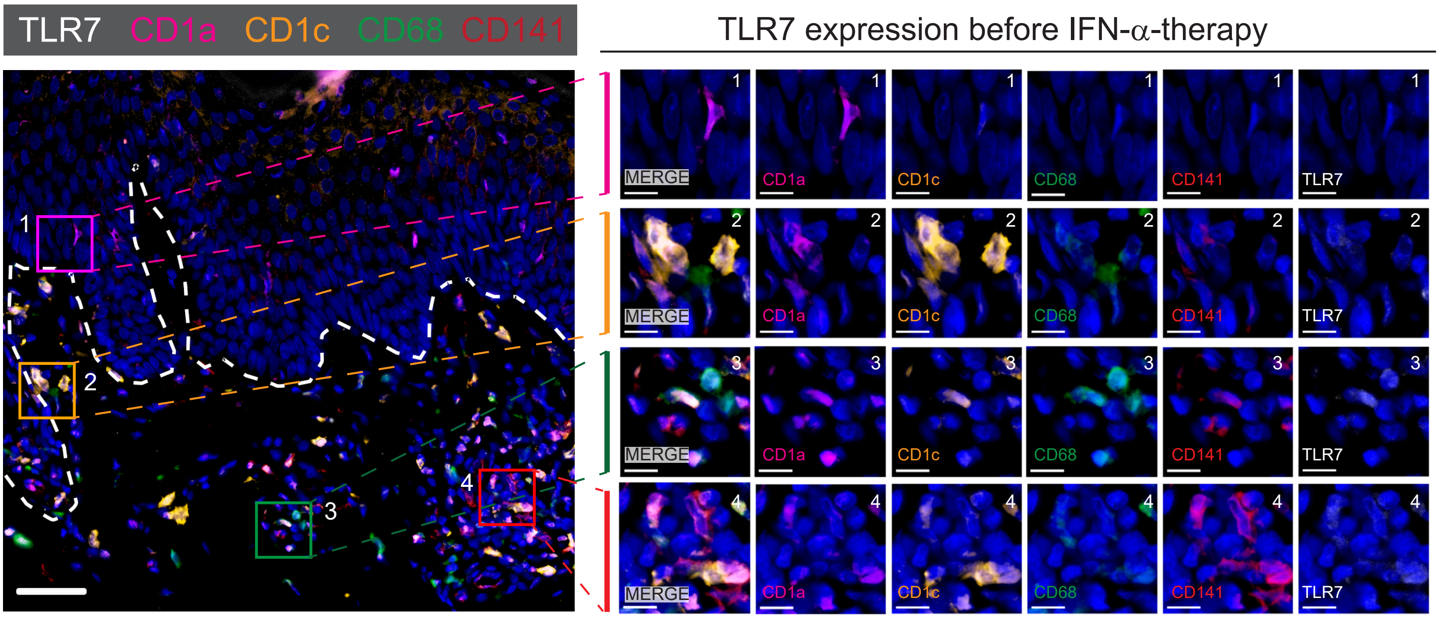

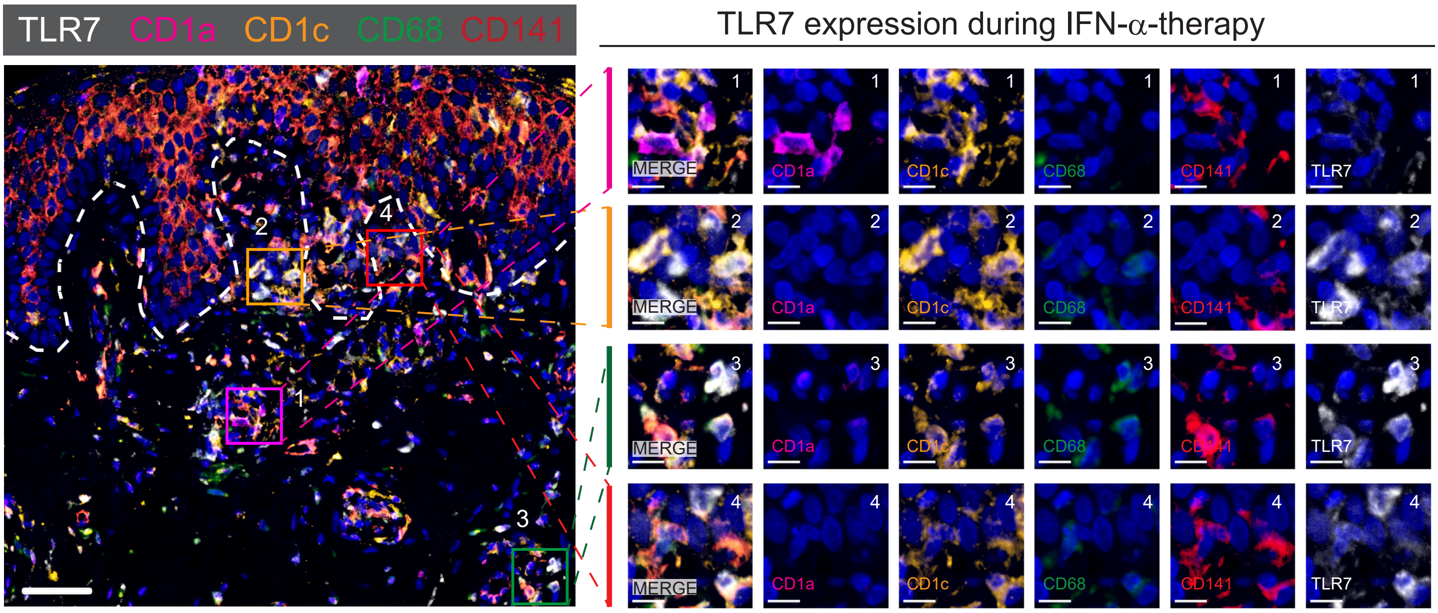

a, Single-color images for each inset are shown for the mulitplex-immunofluorescence (IF) staining described in Extended Data Fig.4a.
