# Supplementary material for: Systemic IFN-I combined with topical TLR7/8 agonists promotes distant tumor suppression by c-Jun-dependent IL-12 expression in dendritic cells
Source: Nat Cancer. 2025 Jan 23;6(1):175–93. doi: 10.1038/s43018-024-00889-9 (PMC11779648; doi:10.1038/s43018-024-00889-9)
Supplement: Supplementary file 2 — Reporting Summary [file 43018_2024_889_MOESM2_ESM.pdf]

nature portfolio

Corresponding author(s): Maria Sibilia

Last updated by author(s): Nov 22, 2024

Reporting Summary

Nature Portfolio wishes to improve the reproducibility of the work that we publish. This form provides structure for consistency and transparency in reporting. For further information on Nature Portfolio policies, see our Editorial Policies and the Editorial Policy Checklist.

Statistics

For all statistical analyses, confirm that the following items are present in the figure legend, table legend, main text, or Methods section.

n/a	Confirmed	
☐	☒	The exact sample size (*n*) for each experimental group/condition, given as a discrete number and unit of measurement
☐	☒	A statement on whether measurements were taken from distinct samples or whether the same sample was measured repeatedly
☐	☒	The statistical test(s) used AND whether they are one- or two-sided<br>*Only common tests should be described solely by name; describe more complex techniques in the Methods section.*
☐	☒	A description of all covariates tested
☐	☒	A description of any assumptions or corrections, such as tests of normality and adjustment for multiple comparisons
☐	☒	A full description of the statistical parameters including central tendency (e.g. means) or other basic estimates (e.g. regression coefficient) AND variation (e.g. standard deviation) or associated estimates of uncertainty (e.g. confidence intervals)
☐	☒	For null hypothesis testing, the test statistic (e.g. *F*, *t*, *r*) with confidence intervals, effect sizes, degrees of freedom and *P* value noted<br>*Give P values as exact values whenever suitable.*
☒	☐	For Bayesian analysis, information on the choice of priors and Markov chain Monte Carlo settings
☒	☐	For hierarchical and complex designs, identification of the appropriate level for tests and full reporting of outcomes
☐	☒	Estimates of effect sizes (e.g. Cohen's *d*, Pearson's *r*), indicating how they were calculated

Our web collection on statistics for biologists contains articles on many of the points above.

Software and code

Policy information about availability of computer code

Data collection

Bioluminescence images were acquired using a Lago X imaging system (Spectral Instruments Imaging).

Microscopy images for IHC were acquired on a Nikon Eclipse 80i microscope with the NIS-Elements Viewer software (v5.22.00) and for multiplex immunofluorescence on a Slide Imaging System (Vectra Polaris, Akoya) with Phenochart Whole Slide Viewer (Akoya, Version 1.1.0) and Inform Tissue Analysis Software (Akoya, Version 2.6).

Western blot images were collected on a ChemiDoc Imaging system (Bio Rad) with Image Lab Software (v6.1).

C1000 Touch Thermal Cycler equipped with a CFX96 Real-Time System (Bio-Rad) was used for qRT-PCR with CFX Maestro Software (v2.3).

Flow cytometry data were collected with BD FACSDIVA software (v9.0; RRID:SCR_001456) or Cytek SpectroFlo (v3.3.0; RRID:SCR_025494).

No custom code was used in this study.

Data analysis

Bioluminescence images were analyzed with the Aura imaging software (v 4.0.8).

Microscopy IHC images were analyzed with ImageJ (v1.53, RRID:SCR_003070; http://imagej.nih.gov/ij) or Adobe Photoshop (RRID:SCR_014199; Adobe Systems, Inc., San Jose, CA). Multiplex immuno-fluorescence images with QuPath (Version 0.4.3) and HALO (indica labs, Version v3.5.3577.214).

Flow cytometry data was analyzed with the FlowJo software (v10.8.1; RRID: SCR_008520).

For the multidimensional tSNE and UMAP analysis, we utilized the following plugins obtained from FlowJo Exchange (https://www.flowjo.com/exchange/#/): DownSample v3.3.1, UMAP v4.0.3 and TriMap v0.2.

For the visualization of flow cytometry data in heatmaps and dot plots, we used the R packages pheatmap (Version: 1.0.12) and ggplot2 (Version: 3.4.3), respectively.

sc-RNA-seq data sets were re-analyzed using the Seurat (v4) R package with the R software (v4.3.1).

Statistical analysis was performed using GraphPad Prism software (v8.0.1).

No custom code was used in this study.

For manuscripts utilizing custom algorithms or software that are central to the research but not yet described in published literature, software must be made available to editors and reviewers. We strongly encourage code deposition in a community repository (e.g. GitHub). See the Nature Portfolio guidelines for submitting code & software for further information.

Data

Policy information about availability of data

All manuscripts must include a data availability statement. This statement should provide the following information, where applicable:

- Accession codes, unique identifiers, or web links for publicly available datasets
- A description of any restrictions on data availability
- For clinical datasets or third party data, please ensure that the statement adheres to our policy

-Accession codes :GSE150361

- Web links:
The Cancer Genome Atlas (TCGA) - SKCM project
http://cancergenome.nih.gov and https://portal.gdc.cancer.gov/

Gene expression - Tlr7
https://www.immune-dictionary.org/app/home
http://rstats.immgen.org/Skyline_microarray/skyline.html?datagroup=IFN

Research involving human participants, their data, or biological material

Policy information about studies with human participants or human data. See also policy information about sex, gender (identity/presentation), and sexual orientation and race, ethnicity and racism.

Reporting on sex and gender	Sex and gender have not been collected.
Reporting on race, ethnicity, or other socially relevant groupings	Race, ethnicity or other socially relevant information have not been collected
Population characteristics	Diagnosis and treatment status (prior or current type I IFN treatment) have been considered in the analysis
Recruitment	Patients samples have been obtained as routine diagnosis and treatment according to national guidelines in the Department of Dermatology of the Klinik Landstrasse (Vienna).
Ethics oversight	All patients signed a written informed consent and did not receive compensation.

Note that full information on the approval of the study protocol must also be provided in the manuscript.

Field-specific reporting

Please select the one below that is the best fit for your research. If you are not sure, read the appropriate sections before making your selection.

☒ Life sciences ☐ Behavioural & social sciences ☐ Ecological, evolutionary & environmental sciences

For a reference copy of the document with all sections, see nature.com/documents/nr-reporting-summary-flat.pdf

Life sciences study design

All studies must disclose on these points even when the disclosure is negative.

Sample size	No statistical methods were used to pre-determine sample sizes but they were chosen to be similar to those reported in previous publications for the same type of experiments (Drobits et al. J Clin Invest 2012). The exact sample sizes (n) are given in the Figure legends.

Data exclusions	Data were excluded if a mathematical outlier was identified using the ROUT (multiple) or Grubbs´ test (one) in GraphPad. Animals were excluded from experiments if they died, or had to be killed to comply to ethical regulations.
Replication	Independent experiments were performed to verify the reproducibility of our experimental findings. The data shown in this study were successfully replicated. The experimental groups (in vitro or in vivo) consisted of n>3 biological replicates.
Randomization	Animals were randomly allocated to experimental groups.
Blinding	The Investigators were not blinded to allocation during experiments and outcome assessment.

Reporting for specific materials, systems and methods

We require information from authors about some types of materials, experimental systems and methods used in many studies. Here, indicate whether each material, system or method listed is relevant to your study. If you are not sure if a list item applies to your research, read the appropriate section before selecting a response.

Materials & experimental systems

n/a	Involved in the study	
☐	☒	Antibodies
☐	☒	Eukaryotic cell lines
☒	☐	Palaeontology and archaeology
☐	☒	Animals and other organisms
☐	☒	Clinical data
☒	☐	Dual use research of concern
☒	☐	Plants

Methods

n/a	Involved in the study	
☒	☐	ChIP-seq
☐	☒	Flow cytometry
☒	☐	MRI-based neuroimaging

Antibodies

Antibodies used

Antibodies for Western blot:
Rabbit anti-mouse c-Jun antibody (Clone 60A8, 9165, Cell Signaling, 1:500) and mouse anti-mouse Vinculin antibody (Clone hVIN-1, V9131, Sigma-Aldrich, 1:500).

Antibodies for IHC:
Goat anti-mouse Endomucin (Thermo Fisher Scientific, 12-5851-80, Polyclonal, pH = 6, 1:200, RRID: AB_2784626), Rabbit anti-mouse CD8a (Abcam, ab217344, EPR21769, pH = 9, 1:500, RRID: AB_2890649), Rabbit anti-human TLR7 (Abcam, ab124928, EPR2088(2), pH = 6, 1:200, RRID: AB_11131208), Goat anti-human TLR8 (Abcam, ab53630, Polyclonal, pH = 6, 1:200, RRID: AB_883061).

Antibodies used for multiplex staining:
Mouse anti-human CD1a (Novus Biologicals, NBP2-34313-0.1mg, O10, pH = 6, 1:200), Mouse anti-human CD1c (LifeTech Austria, TA505411, OTI2F4, pH = 6, 1:200), Mouse anti-human CD68 (Thermo Fisher Scientific, 14-0688-82, KP1, pH = 6, 1:200, RRID), Rabbit anti-human CD141 (Cell Signaling, 43514S, E7Y9P, pH = 6, 1:200), Rabbit anti-human HLA-DR (Thermo Fisher Scientific, MA5-32232, SC06-78, pH = 9, 1:200, RRID: AB_2809518), Rabbit anti-human TLR7 (Abcam, ab124928, EPR2088(2), pH = 6, 1:200, RRID: AB_11131208), Rabbit anti-human XCR1 (Cell Signaling, #44665, D2F8T, pH = 9, 1:100, RRID).

Antibodies for flow cytometry:
anti-mouse CD3 Antibody Alexa Fluor 700 (BioLegend, Cat# 100216, 17A2, 1:200, RRID: AB_493696), anti-mouse CD4 Antibody Pacific Blue (BioLegend, Cat# 100428, GK1.5, 1:200, RRID: AB_493647), anti-mouse CD4 Antibody APC/Cyanine7 (BioLegend, Cat# 100414, GK1.5, 1:200, RRID: RRID:AB_312699), anti-mouse CD4 Antibody Spark Blue 550 (BioLegend, Cat# 100474, GK1.5, 1:200, RRID: RRID:AB_2819768), anti-mouse CD8a Antibody Brilliant Violet 510 (BioLegend, Cat# 100751, 53-6.7, 1:200, RRID: RRID:AB_2563057), anti-mouse CD8a Antibody PE/Cyanine7 (BioLegend, Cat# 100722, 53-6.7, 1:200, RRID: AB_312761), anti-mouse CD8a Antibody APC/Cyanine7 (BioLegend, Cat# 100714, 53-6.7, 1:200, RRID:AB_312753), anti-mouse CD8a Antibody Brilliant Violet 711 (BioLegend, Cat# 100748, 53-6.7, 1:200, RRID:AB_2562100), anti-mouse CD8a Antibody Spark Blue 574 (BioLegend, Cat# 100794, 53-6.7, 1:200, RRID:AB_2922450),anti-mouse CD11b Antibody Brilliant Ultra Violet 563 (BD Biosciences, Cat# 741242, M1/70, 1:200, RRID:AB_2562100), anti-mouse/human CD11b Antibody Brilliant Violet 650 (BioLegend, Cat# 101239, M1/70, 1:200, RRID:AB_11125575), anti-mouse/human CD11b Antibody PE/Cyanine7 (BioLegend, Cat# 101216, M1/70, 1:200, RRID:AB_312799), anti-mouse/human CD11b Antibody PE/Dazzle (BioLegend, Cat# 101255, M1/70, 1:200, RRID:AB_2563647), anti-mouse CD11c Antibody Brilliant Violet 421 (BioLegend, Cat# 117330, N418, 1:200, RRID:AB_11219593), anti-mouse CD11c Antibody Brilliant Ultra Violet 615 (BD Biosciences, Cat# 751222, N418, 1:200, RRID: AB_2875243), anti-mouse CD11c Antibody APC (BioLegend, Cat# 117310, N418, 1:200, RRID:AB_313779), anti-mouse CD11c Antibody PE (BioLegend, Cat# 117307, N418, 1:200, RRID:AB_313776), anti-mouse IL-12/IL-23p40 Antibody PE (BioLegend, Cat# 505204, C15.6, 1:200, RRID:AB_315367), anti-mouse CD11c Antibody PE/Cyanine5 (BioLegend, Cat# 117316, N418, 1:200, RRID:AB_493566), anti-mouse CD19 Antibody Brilliant Violet 750 (BioLegend, Cat# 115561, 6D5, 1:200, RRID:AB_2813978), anti-mouse CD19 Antibody APC/Cyanine7 (BioLegend, Cat# 115529, 6D5, 1:200, RRID:AB_830706), anti-mouse CD19 Antibody PE/Cyanine5 (BioLegend, Cat# 115510, 6D5, 1:200, RRID:AB_313645), anti-mouse CD24 Antibody Pacific Blue (BioLegend, Cat# 101820, M1/69, 1:200, RRID:AB_572011), anti-mouse CD25 Antibody APC/Cyanine7 (BioLegend, Cat# 102026, PC61, 1:200, RRID:AB_830744), anti-mouse CD44 Antibody Pacific Blue (BioLegend, Cat# 103020, IM7, 1:200, RRID:AB_493683), anti-mouse CD45 Antibody APC/Cyanine7 (BioLegend, Cat# 103116, 30-F11, 1:200, RRID:AB_312981), anti-mouse CD45 Antibody Brilliant Violet 510 (BioLegend, Cat# 103138, 30-F11, 1:200, RRID:AB_2563061), anti-mouse CD45 Antibody Brilliant Violet 785 (BioLegend, Cat# 103149, 30-F11, 1:200, RRID:AB_2564590), anti-mouse/human CD45R/B220 Antibody Brilliant

Violet 480 (BD Biosciences, Cat# 565631, RA3-6B2, 1:200, RRID:AB_2739311), anti-mouse/human CD45R/B220 Antibody FITC (BioLegend, Cat# 103206, RA3-6B2, 1:200, RRID:AB_312991), anti-mouse/human CD45R/B220 Antibody APC/Cyanine7 (BioLegend, Cat# 103224, RA3-6B2, 1:200, RRID:AB_313007), anti-mouse CD64 (FcγRI) Antibody Alexa Fluor 488 (BioLegend, Cat# 139316, X54-5/7.1, 1:200, RRID:AB_2566556), anti-mouse CD64 (FcγRI) Antibody PE (BioLegend, Cat# 139304, X54-5/7.1, 1:200, RRID:AB_10612740), anti-mouse CD80 Antibody Alexa Fluor 647 (BioLegend, Cat# 104718, 16-10A1, 1:200, RRID:AB_492824), anti-mouse CD86 Antibody Alexa Fluor 488 (BioLegend, Cat# 105018, GL-1, 1:200, RRID:AB_493463), anti-mouse CD103 Antibody Brilliant Ultra Violet 661 (BD Biosciences, Cat# 750718, 2E7, 1:200, RRID: AB_2874838), anti-mouse CD274 Antibody PE/Cyanine7 (BioLegend, Cat# 124314, 10F.9G2, 1:200, RRID:AB_10643573), anti-mouse CD279 Antibody Brilliant Violet 510 (BioLegend, Cat# 135241, 29F.1A12, 1:200, RRID:AB_2715761), anti-mouse CD317 (BST2, PDCA-1) Antibody Alexa Fluor 488 (biotechne, Cat# FAB8660G, 44E9R, 1:200), anti-mouse CD317 (BST2, PDCA-1) Antibody PE (BioLegend, Cat# 127009, 927, 1:200, RRID:AB_1953284), anti-mouse CD366 Antibody Brilliant Violet 711 (BioLegend, Cat# 119727, RMT3-23, 1:200, RRID:AB_2716208), anti-mouse F4/80 Antibody Alexa Fluor 647 (BioLegend, Cat# 123122, BM8, 1:200, RRID:AB_893480), anti-mouse F4/80 Antibody APC Fire 810 (BioLegend, Cat# 123166, BM8, 1:200, RRID:AB_2894417), anti-mouse Gr-1 Antibody Brilliant Ultra Violet 395 (BD Biosciences, Cat# 563849, RB6-8C5, 1:200, RRID:AB_2738450), anti-mouse I-A/I-E Antibody Brilliant Ultra Violet 496 (BD Biosciences, Cat# 750281, M5/114.15.2, 1:200, RRID:AB_2874472), anti-mouse I-A/I-E Antibody APC (BioLegend, Cat# 107614, M5/114.15.2, 1:200, RRID:AB_313329), anti-mouse I-A/I-E Antibody APC/Cyanine7 (BioLegend, Cat# 107628, M5/114.15.2, 1:200, RRID:AB_2069377), anti-mouse I-A/I-E Antibody Alexa Fluor 700 (BioLegend, Cat# 107622, M5/114.15.2, 1:200, RRID:AB_493727), anti-mouse Ly-6G Antibody Alexa Fluor 700 (BioLegend, Cat# 127622, 1A8, 1:200, RRID:AB_10643269), anti-mouse Ly-6G Antibody Spark YG593 (BioLegend, Cat# 127668, 1A8, 1:200, RRID:AB_2892282), anti-mouse Ly-6C Antibody Pacific Blue (BioLegend, Cat# 128013, HK1.4, 1:200, RRID:AB_1732090), anti-mouse Ly-6C Antibody PE/Cyanine7 (BioLegend, Cat# 128018, HK1.4, 1:200, RRID:AB_1732082), anti-mouse Ly-6C Antibody Brilliant Violet 570 (BioLegend, Cat# 128030, HK1.4, 1:200, RRID:AB_10896061), anti-mouse NK-1.1 Antibody Alexa Fluor 700 (BioLegend, Cat# 108730, PK136, 1:200, RRID:AB_2291262), anti-mouse NK-1.1 Antibody PE/Fire 810 (BioLegend, Cat# 108767, PK136, 1:200, RRID:AB_2936526), anti-mouse NK-1.1 Antibody PE/Cyanine5 (BioLegend, Cat# 108716, PK136, 1:200, RRID:AB_493590), anti-mouse TCR β chain Antibody Brilliant Ultra Violet 737 (eBioscience, Cat# 367-5961-82, H57-597, 1:200, RRID:AB_2896026), anti-mouse TCR β chain Antibody APC (BioLegend, Cat# 109212, H57-597, 1:200, RRID:AB_313435), anti-mouse TCR β chain Antibody APC/Cyanine7 (BioLegend, Cat# 109220, H57-597, 1:200, RRID:AB_893624), anti-mouse TCR β chain Antibody PE (BioLegend, Cat# 109208, H57-597, 1:200, RRID:AB_313431), anti-mouse TCR γ/δ Antibody Alexa Fluor 488 (BioLegend, Cat# 118128, GL3, 1:200, RRID:AB_2562771), anti-mouse TER-119/Erythroid Cells Antibody PE/Cyanine5 (BioLegend, Cat# 116210, Ly-76, 1:200, RRID:AB_313711), anti-mouse TLR7 Antibody PE (BD Biosciences, Cat# 565557, A94B10, 1:200, RRID: AB_2739295), anti-mouse/rat XCR1 Antibody Brilliant Violet 650 (BioLegend, Cat# 148220, ZET, 1:200, RRID:AB_2566410).

For in vivo experiments:
InVivoMAb rat IgG2a isotype control (Clone 2A3 BE0089, Bio X Cell); InVivoMAb anti-mouse IL-12 p40 (Clone 17.8, BE0051, Bio X Cell, 500 µg/mouse); InVivoPlus anti-mouse PD-1 (CD279) (Clone 29F.1A12, BP0273, Bio X Cell, 200 µg/mouse)

Validation

Western blot (anti-mouse c-Jun) :
Cells deficient for c-Jun (genetic knock-out) were used as a negative control.

IHC/multiplex staining:
Specificity of antibodies was assessed with negative controls, where the primary or secondary antibody was omitted on the slide.

Flow cytometry:
Isotype controls and FMO controls (fluorescence minus one) were done to evaluate antibody specificity.
For the anti-mouse TLR7 FACS antibody, we also assessed specificity using a TLR7 knock-out mouse.

Eukaryotic cell lines

Policy information about cell lines and Sex and Gender in Research

Cell line source(s)

The B16-F10 cell line was purchased from the American Type Culture Collection.
The B16-mOVA cell line was a generous gift of Prof. T.F. Tedder (Duke University, USA).
The 4T1 cell line was kindly provided by A. Ciszar (Medical University of Vienna, Austria).

Authentication

Cell lines were authenticated by morphological criteria only. Cells used for experiments were cultured for no more than ten passages.

Mycoplasma contamination

The cell lines were not tested for mycoplasma contamination.

Commonly misidentified lines
(See ICLAC register)

No commonly misidentified cell lines were used.

Animals and other research organisms

Policy information about studies involving animals; ARRIVE guidelines recommended for reporting animal research, and Sex and Gender in Research

Laboratory animals

Female and male mice, 8 to 12 weeks of age, were used in this study.
C57BL/6 were purchased from Harlan Laboratories and BALB/C mice from Janvier Labs.
MMTV-PyMT mice (Strain #:002374, RRID:IMSR_JAX:002374) were purchased from Jackson laboratories.
Tlr7-/- (Hemmi et al., 2002), Ifnar-/- (Müller et al., 1994), Bdca2-DTR (Swiecki and Colonna, 2010), c-Junfl/fl (Behrens et al., 2002) crossed to CD11c-Cre (Caton et al., 2007), or Mx1-Cre (Kühn et al., 1995) were used in this study.

Wild animals | No wild animals were used for this study.

Reporting on sex | Our research findings apply to both sexes.
Except for experiments performed with the genetic breast cancer mouse model (MMTV-PyMT) and the murine 4T1 breast cancer cell line. In these experiments only female mice were used, because tumors normally don´t develop/ grow in male mice. Further information on the sex of our experimental mice has not been collected.

Field-collected samples | No field-collected samples were used in this study.

Ethics oversight | The animal experimental procedures were approved by the Animal Experimental Ethics Committee of the Medical University of Vienna and the Austrian Federal Ministry of Science and Research (Animal license numbers: BMBWF-66.009/0200-WF/II/3b/2014 & BMBWF-66.009/0319-V/3b/2019). Mice were removed from the experiment if the animals displayed any sign of pain, distress, severe weight loss (>20%) or if the tumors were ulcerated or exceeded a tumor volume above 2000mm³. In none of our experiments was this maximal tumor size passed over.

Note that full information on the approval of the study protocol must also be provided in the manuscript.

Clinical data

Policy information about clinical studies
All manuscripts should comply with the ICMJE guidelines for publication of clinical research and a completed CONSORT checklist must be included with all submissions.

Clinical trial registration | *Provide the trial registration number from ClinicalTrials.gov or an equivalent agency.*

Study protocol | *Note where the full trial protocol can be accessed OR if not available, explain why.*

Data collection | *Describe the settings and locales of data collection, noting the time periods of recruitment and data collection.*

Outcomes | *Describe how you pre-defined primary and secondary outcome measures and how you assessed these measures.*

Plants

Seed stocks | -

Novel plant genotypes | -

Authentication | -

Flow Cytometry

Plots

Confirm that:

☒ The axis labels state the marker and fluorochrome used (e.g. CD4-FITC).

☒ The axis scales are clearly visible. Include numbers along axes only for bottom left plot of group (a 'group' is an analysis of identical markers).

☒ All plots are contour plots with outliers or pseudocolor plots.

☒ A numerical value for number of cells or percentage (with statistics) is provided.

Methodology

Sample preparation | Tumors were isolated, cut into pieces and enzymatically digested using 100 µg/mL Liberase (Roche) and 100 µg/mL DNAse I (Sigma) for 45 minutes at 37°C. After digestion, the cells were washed and filtered through a 70 µm cell strainer to obtain a single-cell suspension.

Lymph nodes or spleen were sheard with scissors, incubated for 15 or 30 minutes in a digestion buffer (PBS with Ca2+ and Mg2+) that contained Liberase (100 µg/mL) and DNAse I (100 µg/mL) at 37 °C. To generate a single-cell suspension cells were filtered through a 70 µm cell strainer. Spleen red blood cells were lysed with RBC lysis buffer (BioLegend).

Unspecific binding of antibodies was blocked by incubation of cells with anti-mouse CD16/32 antibody for 10 min at 4°C

	(Clone S17011E, 156604, BioLegend). Subsequently cells were stained with fluorescently labelled antibodies for 30 min at 4°C. Stained cells were washed and afterwards recorded.
Instrument	LSR Fortessa cell analyzer (BD Biosciences, RRID:SCR_018655)<br>FACSAria Fusion Cell Sorter (BD Biosciences, RRID: SCR_018934)<br>Cytek Aurora Spectral Analyzer (Cytek Biosciences, RRID:SCR_019826)
Software	Flow cytometry data was collected with BD FACS DIVA software (v9.0; RRID:SCR_001456)<br>or Cytek SpectroFlo (v3.3.0; RRID:SCR_025494).<br>Flow cytometry data was analyzed with the FlowJo software (v10.8.1; RRID: SCR_008520)
Cell population abundance	Before the relevant cell populations were sorted into Trizol, we performed a test-sort.<br>The purity of this test post-sort fraction had to exceed >90%. for us to continue sorting the samples.
Gating strategy	Our gating strategy to define murine immune cells by flow cytometry started by using a viability dye (7-AAD) to exclude dead cells from the analysis, so that only live cells were considered. Next, to exclude doublets the forward scatter height (FSC-H) to forward scatter area (FSC-A) was compared. Within these cells CD45 identifies immune cells.<br><br>We defined the following immune cell subpopulations in the CD45 gate, as listed below:<br>Lymphoid cells that include B220 and CD19 positive B cells, NK1.1 positive NK cells and TCR-beta and CD3e positive T cells. The T cells were further subdivided in CD4 positive and CD8a positive subsets. CD8a T cells were analyzed for the activation/ exhaustion markers CD44, PD-1 and TIM-3.<br><br>Myeloid cells that include Ly-6G and CD11b positive Neutrophils, CD64, as well as F4/80 and CD11b positive Macrophages and Ly-6C and CD11b positive Monocytes were analyzed. Lastly, dendritic cells were defined by the expression of the surface markers CD11c and MHC-II, and the absence of the marker CD64. Type-I dendritic cells were gated as positive for XCR1, whereas Type-II dendritic cells were gated as CD11b positive among the dendritic cells defined. Plasmacytoid dendritic cells were gated as BST-2, B220 positive cells that showed intermediate expression of CD11c and were negative for CD11b. Dendritic cell subsets were further analyzed for the expression of the activation/exhaustion markers CD80, CD86 and PD-L1.<br><br>Please refer to Extended Data Figure 6 a, e and Extended Data Figure 9a.

☒ Tick this box to confirm that a figure exemplifying the gating strategy is provided in the Supplementary Information.
