# Supplementary material for: Systemic IFN-I combined with topical TLR7/8 agonists promotes distant tumor suppression by c-Jun-dependent IL-12 expression in dendritic cells
Source: Nat Cancer. 2025 Jan 23;6(1):175–93. doi: 10.1038/s43018-024-00889-9 (PMC11779648; doi:10.1038/s43018-024-00889-9)

Source Data for Extended Data Figure 8

Extended Data Fig 8b - Unprocessed blots

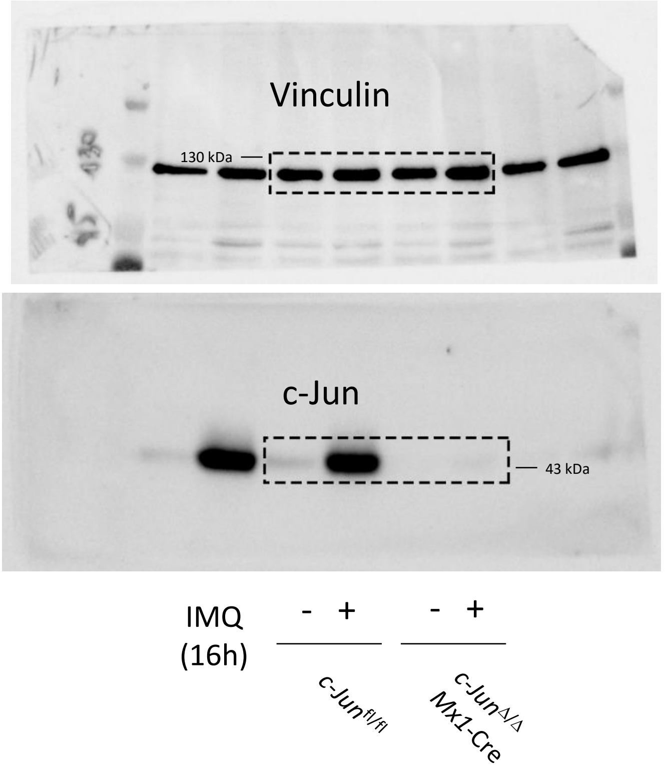

Extended Data Fig 8b – Protein ladder

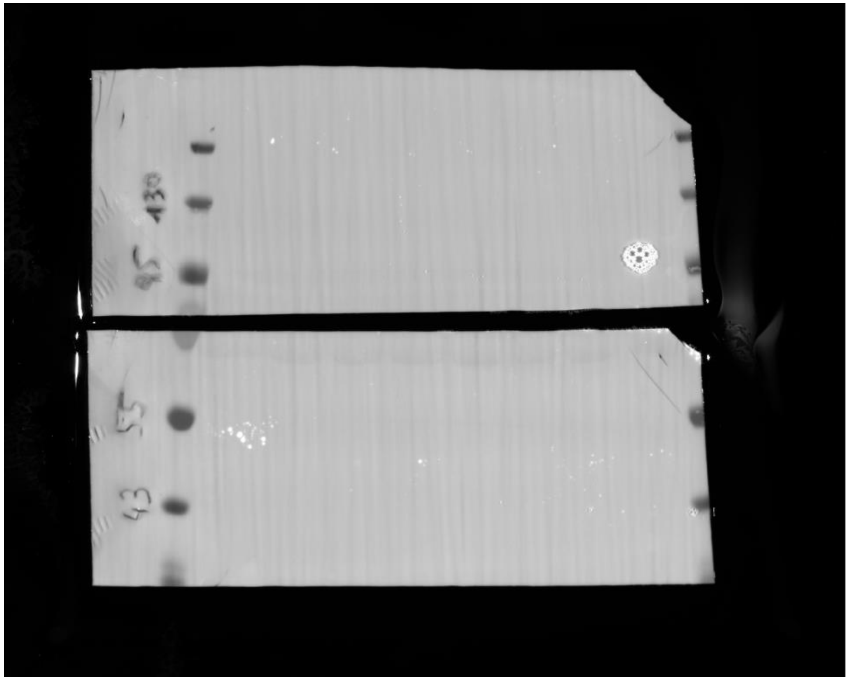

Supplement: Supplementary file 21 — Unprocessed blots. [file 43018_2024_889_MOESM21_ESM.pdf]
